# Functional Characterization and Categorization of Missense Mutations that Cause Methylmalonyl‐CoA Mutase (MUT) Deficiency

**DOI:** 10.1002/humu.22633

**Published:** 2014-11-24

**Authors:** Patrick Forny, D. Sean Froese, Terttu Suormala, Wyatt W. Yue, Matthias R. Baumgartner

**Affiliations:** ^1^ Division for Metabolic Disorders and Children's Research CenterUniversity Children's HospitalZurichSwitzerland; ^2^ Zurich Center for Integrative Human PhysiologyUniversity of ZurichSwitzerland; ^3^ Structural Genomics ConsortiumUniversity of OxfordUK

**Keywords:** methylmalonic aciduria, methylmalonyl‐CoA mutase, MUT, cobalamin, thermolability

## Abstract

Methylmalonyl‐CoA mutase (MUT) is an essential enzyme in propionate catabolism that requires adenosylcobalamin as a cofactor. Almost 250 inherited mutations in the *MUT* gene are known to cause the devastating disorder methylmalonic aciduria; however, the mechanism of dysfunction of these mutations, more than half of which are missense changes, has not been thoroughly investigated. Here, we examined 23 patient missense mutations covering a spectrum of exonic/structural regions, clinical phenotypes, and ethnic populations in order to determine their influence on *protein stability*, using two recombinant expression systems and a thermostability assay, and *enzymatic function* by measuring MUT activity and affinity for its cofactor and substrate. Our data stratify MUT missense mutations into categories of biochemical defects, including (1) reduced protein level due to misfolding, (2) increased thermolability, (3) impaired enzyme activity, and (4) reduced cofactor response in substrate turnover. We further demonstrate the stabilization of wild‐type and thermolabile mutants by chemical chaperones in vitro and in bacterial cells. This in‐depth mutation study illustrates the tools available for MUT enzyme characterization, guides future categorization of further missense mutations, and supports the development of alternative, chaperone‐based therapy for patients not responding to current treatment.

## Introduction

Mitochondrial methylmalonyl‐CoA mutase (MUT, EC 5.4.99.2) catalyzes the reversible isomerisation of l‐methylmalonyl‐CoA to succinyl‐CoA, requiring vitamin B_12_ (cobalamin) in the form of adenosylcobalamin (AdoCbl) as a cofactor. In humans, this reaction represents an important step in propionate catabolism, funneling metabolites from the breakdown of amino acids (valine, isoleucine, methionine, and threonine), odd‐chain fatty acids, and the side chain of cholesterol into the tricarboxylic acid cycle. The MUT enzyme is highly conserved from bacteria to human and is well studied at the enzymatic [Fenton et al., 1982; Banerjee and Ragsdale, 2003] and structural level [Mancia et al., 1996; Froese et al., 2010b]. The importance of the MUT‐catalyzed reaction is further underlined by the metabolic disorder methylmalonic aciduria (MMA), which is caused by a genetic defect in the MUT enzyme itself (MIM# 251000, MMA *mut* type), or in one of several proteins (MMAA, MMAB, MMADHC) involved in the uptake, modification, and delivery of AdoCbl to the MUT enzyme for its activity (MMA *cblA* type, MIM# 251100; MMA *cblB* type, MIM# 251110; MMA *cblD‐variant 2* MIM# 277410) [Fowler et al., 2008].

The human *MUT* gene (MIM# 609058; chromosome location 6p12–21.2) [Ledley et al., 1988a, b] is the site of almost 250 deleterious mutations reported to cause MMA [Froese and Gravel, 2010] (human gene mutation database, HGMD Professional version as of December 2013, hgmd.org). Patient cell lines can be assigned to the *mut‐*type MMA by complementation analysis of fibroblast heterokaryons [Gravel et al., 1975; Willard et al., 1978]. Further biochemical characterization, applying MUT activity assay and propionate fixation, allows *mut* classification into two subtypes. Mutants with residual mutase activity in cell homogenates under saturating concentrations of AdoCbl, and whose ability to incorporate [1–^14^C]propionate into acid precipitable material in intact skin fibroblasts is responsive to supplementation of the culture medium with hydroxocobalamin, are designated *mut* ^−^; mutants with no residual MUT activity and no response of propionate incorporation to hydroxocobalamin are designated *mut* ^0^ [Willard and Rosenberg, 1980]. *mut* ^0^ patients often present in the newborn period with ketoacidosis, lethargy, repeated vomiting, coma or even death, and suffer from severe long‐term complications such as renal failure and neurological impairments. On the contrary, *mut* ^−^ patients have a lower occurrence of mortality, morbidity, and long‐term complications [Horster et al., 2007]. Dietary interventions, carnitine supplementation, and symptom management are the mainstay of current treatment, although pharmacological doses of hydroxocobalamin are given to some MUT‐deficient patients [Horster et al., 2007]. Although some *mut* ^−^ patients might respond to this treatment, others—including most if not all *mut* ^0^ patients—do not [Horster et al., 2007]. Alternative therapeutic approaches should therefore be investigated, evaluating therapeutic potential on the basis of a systematic characterization of individual *mut* ^0^ and *mut* ^−^ alleles. Nevertheless, the sheer quantity of mutations in the *MUT* gene—most of which are private and rare [Acquaviva et al., 2005; Worgan et al., 2006; Lempp et al., 2007; Fowler et al., 2008]—means that such a large‐scale analysis has not been forthcoming, with previous reports of in vitro MUT characterization on only a handful of mutations [e.g., Crane and Ledley, 1994; Janata et al., 1997].

Common to many other metabolic disorders (e.g., phenylketonuria (PKU) [Mitchell et al., 2011]; ornithine transcarbamylase deficiency [Shchelochkov et al., 2009]), the largest proportion of *MUT* mutations are missense changes (131 of 243, 54%; hgmd.org) whose effects on the protein are difficult to predict a priori [Froese and Gravel, 2010; Yue et al., 2014]. MUT is therefore an attractive target to interrogate the genotype‐specific biochemical penalties for missense mutations at the protein level, an approach adopted for other metabolic enzymes [Pey et al., 2007; Pekkala et al., 2010; Shi et al., 2012]. With this in mind, we have chosen seven *mut* ^0^ and 16 *mut* ^−^ missense mutations for an in‐depth analysis. On the basis of recombinant expression (*Escherichia coli* and human fibroblasts), thermal denaturation, and enzyme assays, we catalogue each mutation as defective in stability, activity, or both, and further define subcategories in each. To the best of our knowledge, this work represents the first large‐scale biochemical categorization of MUT mutations and sets the stage for exploring the potential of small molecule therapeutics, an approach that is gaining wide interest to augment mutant enzyme activity [Gomes, 2012].

## Materials and Methods

### Cloning

For expression in *E. coli*, we used a previously described construct of MUT (called hMUT in [Froese et al., 2010b]). For expression in fibroblasts, the MUT cDNA sequence was purchased from GeneCopoeia (Rockville, MD, USA) (Construct ID: E0205; RefSeq NM_000255.3) and subsequently subcloned into pTracer‐CMV2 (Invitrogen, Carlsbad, CA, USA) via *Eco*RV and *Not*I restriction sites by PCR using the following primers: ACATAGATATCCACGCTGTTTTGA (*Eco*RV site underlined) and AGTGGTTGATCGCGTGCATG (*Not*I site is part of the original GeneCopoeia plasmid). All single‐site missense mutations were generated using the QuikChange site‐directed mutagenesis kit (Stratagene, La Jolla, CA, USA) following manufacturer's instructions, using forward and reverse primers (Microsynth, Balgach, Switzerland) described in Supp. Table S1, and confirmed by Sanger sequencing.

### Bacterial Expression and Purification

MUT was expressed in *E. coli* and purified as previously described [Froese et al., 2010b] with minor modifications. For small‐scale purification, cells were grown in a total of 50 ml, induced with 0.1 mM isopropyl β‐D‐1‐thiogalactopyranoside (IPTG) at 18°C overnight, harvested by centrifugation at 4,000*g*, lysed by sonication, and purified by affinity (Ni‐NTA; Qiagen, Venlo, The Netherlands) chromatography. Where applicable, chemical chaperones were added concurrently with the IPTG. Samples from total cell lysate (1 μl of 2 ml total) (“L”), including all cellular proteins both soluble and insoluble, and affinity eluants (15 μl of 250 μl total) (“E”), including those soluble proteins eluted from the nickel affinity column, were analyzed by SDS‐PAGE and stained with Coomassie blue (Expedeon, San Diego, CA, USA). For large‐scale purification, cells were grown in a total of 6 l, harvested by centrifugation at 5,000*g*, lysed with an Emulsiflex C3 homogenizer, and purified by affinity (Ni‐NTA, Qiagen), size‐exclusion (Superdex 200; GE Healthcare, Little Chalfont, UK), cleavage with His‐tagged TEV protease and reverse‐affinity chromatography (Ni‐NTA). Purification buffers are detailed in the webpage http://www.thesgc.org/structures/details?pdbid=2XIQ.

### Transfection and Expression in Fibroblasts

Fibroblasts of a *mut* ^0^ patient homozygous for the mutation p.Q30* were immortalized by transfection with pRNS1 [Litzkas et al., 1984] using electroporation [Baumgartner et al., 2001] and grown in Dulbecco's Modified Eagle Medium (Gibco, Life Technologies, Zug, Switzerland) supplemented with 10% fetal bovine serum (Gibco) and antibiotics (GE Healthcare), as previously described [Suormala et al., 2004]. pTracer‐MUT wild‐type (*wt*) and mutant constructs were transiently transfected into the immortalized fibroblasts by electroporation with a transfection efficiency of 6%–24%, as determined by fluorescence‐associated cell sorting measuring the proportion of cells coexpressing the green fluorescent protein from the pTracer construct. This is comparable to previous observations [Coelho et al., 2008]. Cells were harvested 48 hr after electroporation by trypsinization, washed twice with phosphate buffered saline, and stored frozen at −80°C until assayed for MUT activity or used for Western blotting.

### Western Blot

Fibroblast lysates were obtained by sonication (details under MUT activity assay). Crude cell lysates (25 μg of protein per sample) were mixed with 2× Laemmli Sample Buffer (Bio‐Rad, Hercules, CA, USA) and heated to 96°C for 6 min. Proteins were separated by 10% SDS‐PAGE, transferred to a Protran BA85 nitrocellulose membrane (Whatman, GE Healthcare), blocked at room temperature for 1 hr with buffer A (5% skimmed milk, 1.2% w/v Tris‐base, 9% w/v NaCl, 0.2% Tween 20, pH 7.6), incubated with polyclonal mouse anti‐human MUT (Abcam, Cambridge, UK) (1:1,000 in buffer A), or β‐actin (Sigma–Aldrich, Buchs SG, Switzerland) (1:4,000 in buffer A) overnight, detected by incubation with goat anti‐mouse antibody coupled to horseradish peroxidase (Santa Cruz, Biotechnology, Dallas, TX, USA) (1:5,000 in buffer A) for 1 hr, and visualized with ECL Western Blotting Detection Reagents (GE Healthcare) on a Gel Logic 6000 Pro (Carestream, Gland, Switzerland).

### MUT Activity Assay

MUT activity was assayed in crude cell lysates by measuring the production of [^14^C]succinate from [^14^C]methylmalonyl‐CoA by a modification of earlier described methods [Baumgartner, 1983; Causey and Bartlett, 1984]. All operations were performed in a dark room illuminated with a 15 W red Safelight bulb (Dr. Fischer, Diez/Lahn, Germany). Cell lysates were prepared by pipetting 5 mM potassium phosphate buffer (pH 7.4) on frozen cells and disrupting the pellet by sonicating twice for 15 sec using the microprobe of an XL‐2000 sonicator (Microson, Qsonica, Newtown, CT, USA) at amplitude 1.5. Specific activities were measured in 50 μl reaction mixture containing 0.1 M potassium phosphate buffer (pH 7.4), 1 mM DL‐2‐[methyl‐^14^C]methylmalonyl‐CoA (American Radiolabeled Chemicals (ARC), St. Louis, MO, USA; specific activity 7.03 MBq/mmol in assay), and 50–100 μg cell proteins without (*holo*‐MUT activity) and with (total MUT activity) 50 μM AdoCbl. To estimate *K*_M_ for AdoCbl, its concentration was varied between 0.0025 and 50 μM. To estimate *K*_M_ for methylmalonyl‐CoA, its concentration was varied between 0.01 and 1.0 mM in the presence of 50 μM AdoCbl. Reactions were initiated by the addition of cell lysate, allowed to proceed for 30 min at 37°C, and terminated by addition of 6 μl 5 N KOH (Merck, Whitehouse Station, NJ, USA). Samples were reincubated for 15 min at 37°C to hydrolyze CoA derivatives, neutralized by adding 5 μl 5 N perchloric acid (Merck), and spiked with 5 μl of 1% solution (w/v) of succinic acid (Merck) in order to visualize succinate during HPLC separation. Samples were centrifuged to remove precipitate, 50 μl of the supernatant was injected into an Amimex HPX‐87H Ion Exclusion column (300 × 7.8 mm^2^; H‐form, 9 μm, Bio‐Rad), and [^14^C]succinate was separated from [^14^C]methylmalonate by elution with 0.5 mM H_2_SO_4_ at 30°C using a flow rate of 0.4 ml/min. Succinate (retention time 17 min) and methylmalonate (11 min) peaks were visualized at 210 nm using a UV detector; 0.2 ml fractions covering the succinate peak were collected and [^14^C]succinate was quantitated in a Tri‐Carb C1 900TR scintillation spectrometer (PerkinElmer, Waltham, MA, USA) with Optiphase HiSafe 2 counting cocktail (PerkinElmer). Protein in cell lysates was determined by the Lowry method. MUT activity was expressed as nanomole succinate formed per minute and milligram of protein. *K*_M_ values for AdoCbl were determined using Eadie–Hofstee plotting.

### Differential Scanning Fluorimetry

Purified homodimeric MUT was assayed for shifts in melting temperature (*T*_m_), which is the midpoint transition from a folded to an unfolded state, as previously described [Niesen et al., 2007; Froese et al., 2010a]. Each mutant and *wt* protein was assayed in the *apo* state, or with preincubation of 50 μM AdoCbl (Sigma–Aldrich), or a combination of 50 μM AdoCbl and 500 μM malonyl‐CoA (MCoA; Sigma–Aldrich). MCoA was used as a substrate analogue because it has been confirmed to bind to the substrate pocket (PDB: 2XIQ) [Froese et al., 2010b]. For chemical osmolyte screening, various concentrations of proline, betaine, sorbitol, TMAO, sucrose (up to 1,200 mM), and glycerol (up to 20%) were preincubated with protein before analysis.

## Results

### Rationale for Mutation Selection

From the repertoire of known *MUT* missense mutations, we selected 23 for this study (Table 1) with the rationale that they (1) cover both phenotypically severe *mut* ^0^ (*n* = 7) and milder *mut* ^−^ (*n* = 16) subtypes, (2) are widely distributed across the gene (exons 2–13), and (3) include representative mutations prevalent in different ethnic populations, for example, African Americans (p.G717V and c.2150G>T) [Worgan et al., 2006], Caucasians (p.N219Y, c.655A>T; p.R369H, c. 1106G>A; p.R694W, c.2080C>T) [Acquaviva et al., 2005; Lempp et al., 2007], and Turkish Asians (p.P615T, c.1843C>A) [Dundar et al., 2012]. These 23 mutations, reported in both homozygous and heterozygous states, are found in highly conserved residues, and their amino acid substitutions are predicted to be largely untolerated according to *SIFT* prediction (Table 1B). All selected mutations have been reported (Table 1A, references therein) with the exception of p.L736F, c.2206C>T, a novel mutation that exists in a compound heterozygous state with the splice site mutation c.753+2T>A [Worgan et al., 2006] in an MMA patient diagnosed in our laboratory. Propionate fixation and MUT activity assays on cells from this patient are consistent with the *mut* ^−^ subtype.

**Table 1:** Selection of Mutations in the *MUT* Gene and Study Results

Prestudy data					Study results			
Predicted amino acid change	Nucleotide change^a^	Genomic location	*mut* subtype	References	Category of biochemical defect	Conservation score^b^	Buried surface at dimer (%)^c^	SIFT tolerance^d^
p.P86L	c.257C>T	Exon 2	*mut* ^−^	[Worgan et al., 2006]	Thermolabile, *K*_M_	0.705	100	0.00
p.Y100C	c.299A>G	Exon 2	*mut* ^−^	[Lempp et al., 2007]	*K*_M_	0.953	0	0.00
p.A191E	c.572C>A	Exon 3	*mut* ^0^	[Acquaviva et al., 2005; Worgan et al., 2006]	Folding, catalytic	0.936	0	0.00
p.Q218H	c.654A>C	Exon 3	*mut* ^0^	[Worgan et al., 2006]	Catalytic	1.000	0	0.00
p.N219Y	c.655A>T	Exon 3	*mut* ^0^	[Acquaviva et al., 2005; Worgan et al., 2006; Lempp et al., 2007]	Catalytic	1.000	0	0.00
p.Y231N	c.691T>A	Exon 3	*mut* ^−^	[Worgan et al., 2006; Lempp et al., 2007]	*K*_M_	0.951	0	0.00
p.Y316C	c.947A>G	Exon 5	*mut* ^−^	[Worgan et al., 2006]	Unclear	0.817	97	0.00
p.L328F	c.982C>T	Exon 5	*mut* ^0^	[Acquaviva et al., 2005; Lempp et al., 2007]	Folding, catalytic	0.901	0	0.01
p.S344F	c.1031C>T	Exon 5	*mut* ^−^	[Lempp et al., 2007]	Folding, catalytic	0.944	0	0.00
p.N366S	c.1097A>G	Exon 6	*mut* ^−^	[Lempp et al., 2007]	Catalytic	1.000	0	0.00
p.R369H	c.1106G>A	Exon 6	*mut* ^0^	[Worgan et al., 2006; Lempp et al., 2007]	Catalytic	1.000	99	0.00
p.T387I	c.1160C>T	Exon 6	*mut* ^−^	[Dundar et al., 2012]	Unclear	1.000	0	0.00
p.G426R	c.1276G>A	Exon 6	*mut* ^−^	[Worgan et al., 2006]	Thermolabile, *K*_M_	0.702	98	0.00
p.F573S	c.1718T>C	Exon 10	*mut* ^−^	[Worgan et al., 2006]	Folding	0.586	0	0.03
p.P615T	c.1843C>A	Exon 11	*mut* ^0^	[Acquaviva et al., 2005; Lempp et al., 2007]	Folding	0.862	0	0.00
p.P615L	c.1844C>T	Exon 11	*mut* ^0^	[Dundar et al., 2012]	Folding	0.862	0	0.00
p.V633G	c.1898T>G	Exon 11	*mut* ^−^	[Worgan et al., 2006; Lempp et al., 2007]	*K*_M_	0.794	0	0.00
p.G648D	c.1943G>A	Exon 11	*mut* ^−^	[Ledley and Rosenblatt, 1997]	*K*_M_	0.729	0	0.00
p.R694W	c.2080C>T	Exon 12	*mut* ^−^	[Acquaviva et al., 2005; Lempp et al., 2007]	Catalytic	0.549	0	0.00
p.R694L	c.2081G>T	Exon 12	*mut* ^−^	[Lempp et al., 2007]	Thermolabile, catalytic	0.549	0	0.08
p.M700K	c.2099T>A	Exon 12	*mut* ^−^	[Acquaviva et al., 2005; Worgan et al., 2006; Lempp et al., 2007]	Catalytic	0.570	0	0.00
p.G717V	c.2150G>T	Exon 13	*mut* ^−^	[Worgan et al., 2006]	Thermolabile, *K*_M_	0.840	0	0.00
p.L736F	c.2206C>T	Exon 13	*mut* ^−^	This study^e^	Unclear	0.625	0	0.02

a Nucleotide numbering uses +1 as the A of the ATG translation initiation codon in the reference sequence (NM_000255.3), with the initiation codon as codon 1.

b The conservation score (0 = unconserved; 1 = strictly conserved) was calculated using Scorecons server (http://www.ebi.ac.uk/thornton‐srv/databases/cgi‐bin/valdar/scorecons_server.pl, as of June 2014) [Valdar, 2002], based on a multiple sequence alignment of 146 MUT homologues from different phyla, generated from the CONSURF server (http://consurf.tau.ac.il/, as of June 2014) [Ashkenazy et al., 2010].

c Percentage of the amino acid total surface that is buried in the human MUT dimer, calculated from the PISA server v1.48 (http://www.ebi.ac.uk/pdbe/pisa/) [Krissinel and Henrick, 2007].

d SIFT tolerance score (0–0.05 corresponds to amino acid changes that likely affect protein function) was calculated using the SIFT server (http://sift.jcvi.org/, as of June 2014) [Kumar et al., 2009].

e The new variant has been submitted to dbSNP (http://www.ncbi.nlm.nih.gov/SNP/).

**Figure 1:**
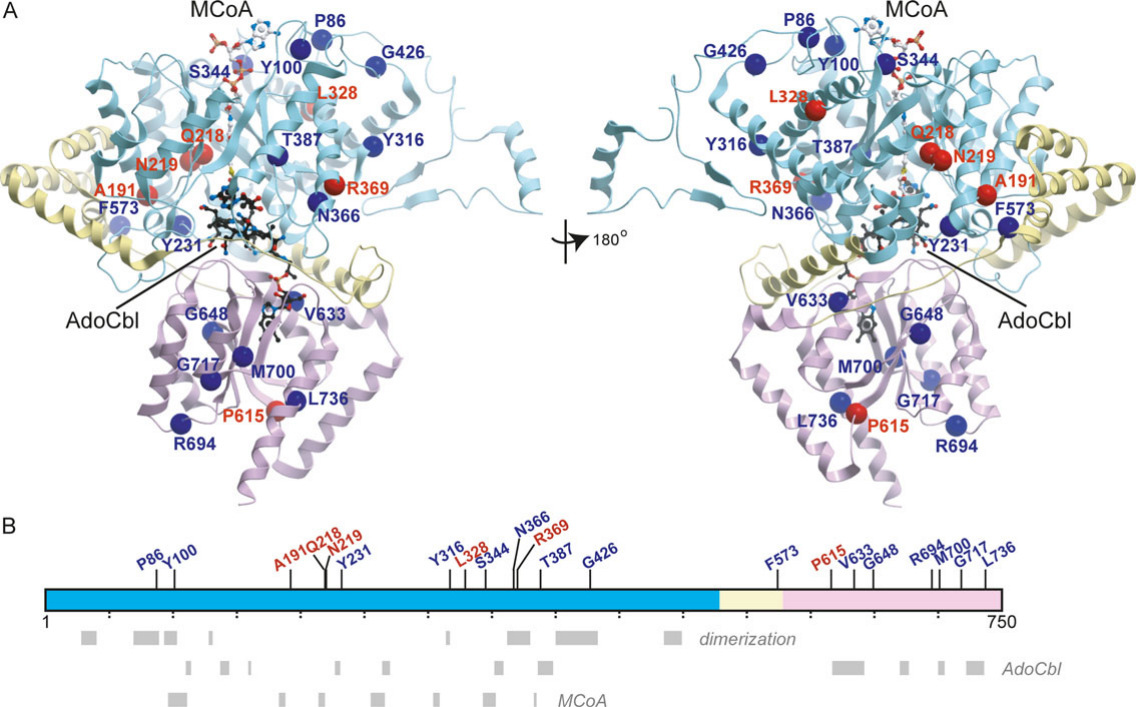
Structural mapping of MUT missense mutations. **A:** The human MUT structure in complex with MCoA and AdoCbl (PDB code 2XIQ) is colored according to domains, that is, N‐terminal substrate‐binding domain cyan, C‐terminal cofactor‐binding domain magenta, and interdomain linker yellow. Mutations in this study are shown as red circles (*mut* ^0^) or blue circles (*mut* ^−^). Ligands are in stick representation, colored white for MCoA and black for AdoCbl. **B:** Domain organization of MUT highlighting locations of the studied mutations, dimerization interface, and binding regions for MCoA and AdoCbl in the polypeptide. An interactive version of this structural representation is available online at www.thesgc.org/MUT.

We mapped the missense mutations onto the human MUT crystal structure [Froese et al., 2010b] to understand their local structural environments (Supp. Fig. S1; an interactive version is available at www.thesgc.org/MUT). These mutations are located across the entire polypeptide, including the N‐terminal substrate‐binding domain (*n* = 13), C‐terminal cofactor‐binding domain (*n* = 9), and interdomain linker (*n* = 1) (Fig. 1A). A number of them contribute to the binding regions of the substrate (e.g., p.Y100C) and cofactor (e.g., p.M700K) (Fig. 1B), whereas four residues are buried at, or located near, the MUT dimer interface (p.P86L, p.Y316C, p.R369H, p.G426R) (Table 1B and Fig. 1B). Compatible with the large proportion of private mutations, no hot spot regions are found for the above, or other known MUT mutations.

### Effects on Protein Integrity in Two Expression Systems

**Figure 2:**
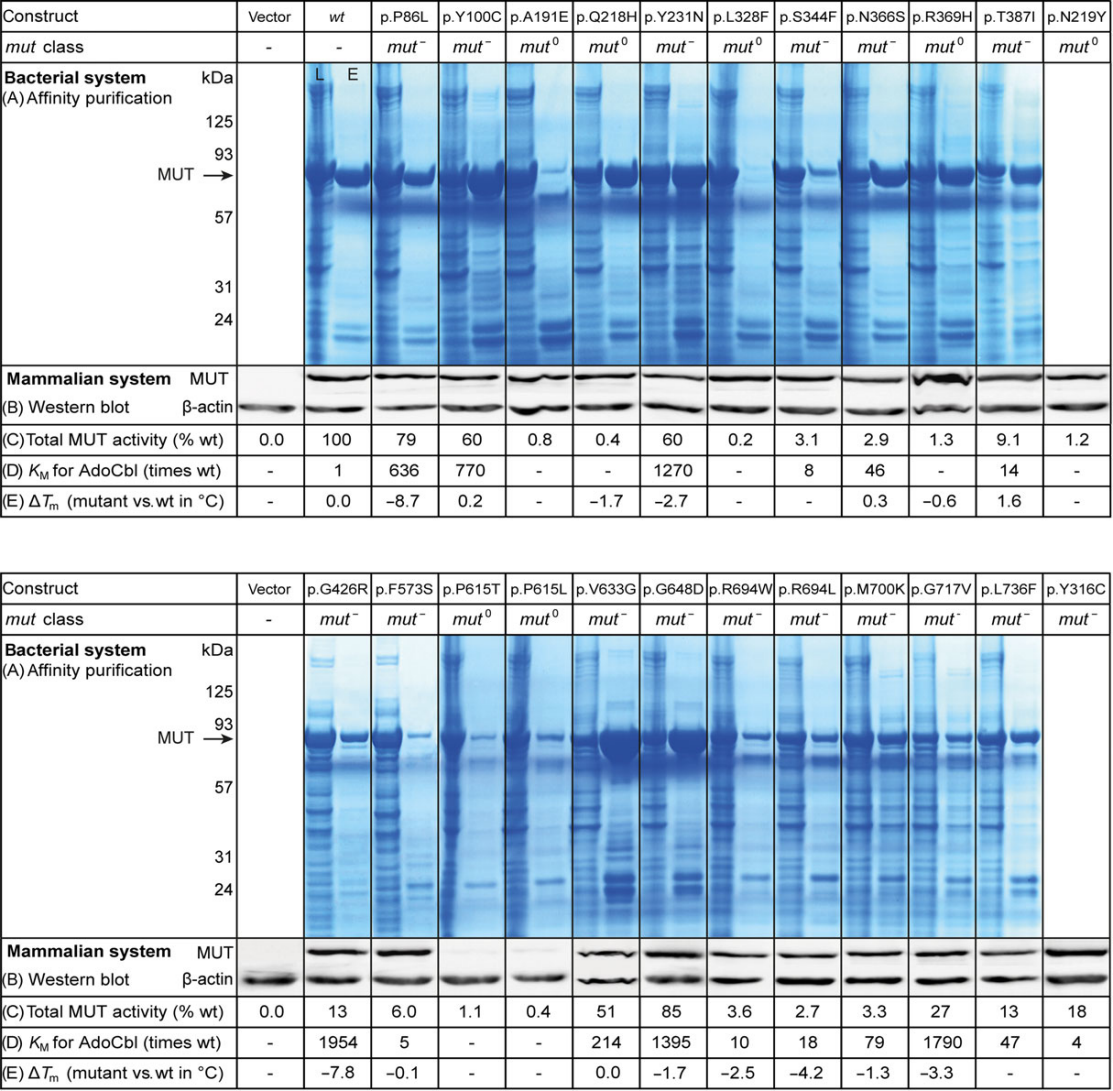
MUT missense mutations confer different effects on stability and activity. **A:** Coomassie staining of SDS‐PAGE following small‐scale bacterial expression and affinity purification. For each mutation, lanes for total cell lysate (“L,” left) and eluant after purification (“E,” right) are shown. **B:** Western blot following expression of each mutation in a MUT‐deficient patient cell line. Vector, empty vector. **C:** Total MUT activity (assay with 50 μM AdoCbl) of each mutation expressed as percent of mean *wt* activity. **D:** *K*_M_ for AdoCbl, expressed as times the mean *wt* value. **E:** ∆*T*_m_ of *apo* mutant compared to *apo‐wt* protein (see Fig. 3). –, not applicable.

The diverse structural locations of the 23 missense mutations, many of which are distant from the catalytic center, suggest some mutations may impact on noncatalytic properties such as the stability of translated polypeptides. To investigate this, we reconstructed these alleles in two recombinant expression systems, *E. coli* and human fibroblasts (Fig. 2A and B), both of which have been used to produce MUT previously [Wilkemeyer et al., 1991; Janata et al., 1997; Froese et al., 2010b]. For *E. coli* expression, the mutant proteins (*n* = 21, excluding p.N219Y and p.Y316C that we were unable to clone) were assessed on their (1) expression level relative to *wt* protein, as judged by protein amount in total cell lysate, and (2) solubility, as judged by protein amount after affinity purification. We first tested several postinduction growth temperatures (37/26/18/12°C) for *wt* MUT and observed the highest solubility level with 18°C overnight growth (data not shown). Although all 21 mutants are expressed in *E. coli* at this temperature (Fig. 2A, lanes “L”), a number of them have poor solubility compared with *wt*, resulting in no (p.A191E and p.L328F) or reduced (p.S344F, p.F573S, p.P615T/L) protein recovery from the affinity eluant (lanes “E”). This observation, unchanged by a lower postinduction temperature (12°C; data not shown), indicates insoluble mutant protein expression likely due to a global folding defect. In addition to *E. coli*, we expressed MUT mutants using an immortalized MUT‐deficient patient fibroblast cell line. Western blot (Fig. 2B) of the expressed mutants detected very low protein levels for p.P615T/L, which agrees with bacterial expression. However, the p.A191E and p.L328F proteins, insoluble in *E. coli*, have near‐*wt* expression levels in human fibroblasts, suggesting that additional factors exclusive to eukaryotes may assist in the proper folding for certain mutant proteins.

### Effects on Enzymatic Function

MUT catalyzes the isomerization of methylmalonyl‐CoA to succinyl‐CoA via reactive radical intermediates generated from a homolytic bond cleavage of AdoCbl [Banerjee and Ragsdale, 2003]. To investigate if and how each mutation affects MUT enzymatic function, we determined catalytic activity and ligand affinity for *wt* and mutant MUT expressed in MUT‐deficient human fibroblasts. Under saturating AdoCbl levels (50 μM), *wt* MUT exhibited a mean substrate turnover of 20.2 nmol/min/mg protein (Supp. Table S2). All 23 mutants showed decreased enzyme activity, ranging from 0.2% to 85% of *wt* values (Fig. 2C; Supp. Table S2). They can be broadly described as having *high* (>50% of *wt*, *n* = 5), *intermediate* (5%–50% of *wt*, *n* = 6), and *low* (<5% of *wt*, *n* = 12) levels of residual enzyme activity (Supp. Fig. S2). All *mut* ^0^ mutants studied were near the assay detection limit (<2% of *wt*), consistent with their reported clinical severity, whereas the *mut* ^−^ mutants (2.7%–85% of *wt*) are distributed into all three levels of activity. The more substantial residual activity of *mut^−^* mutations allowed us to further determine their *K*_M_ values for AdoCbl and methylmalonyl‐CoA. *wt* MUT had a mean *K*_M_ for AdoCbl of 4.7 nM (Supp. Table S2), similar to published values [Morrow et al., 1978]. All characterized mutants exhibited higher *K*_M_ values than *wt*, to varying degrees (Fig. 2D). Seven proteins had a *K*_M_ of >200 times *wt* levels, of which four (p.Y231N, p.G426R, p.G648D, and p.G717V) are at least three orders of magnitude higher, indicating a strong *K*_M_ defect. On the contrary, five proteins had near‐*wt K*_M_ (e.g., Y316C and p.F573S, <5 times *wt*), suggesting their catalytic impairment is not primarily due to reduced cofactor affinity. No mutants showed large increases in *K*_M_ for methylmalonyl‐CoA (data not shown), suggesting substrate binding was not defective in these mutants, as has been shown previously [Willard and Rosenberg, 1980].

### Effects on Protein Thermolability

**Figure 3:**
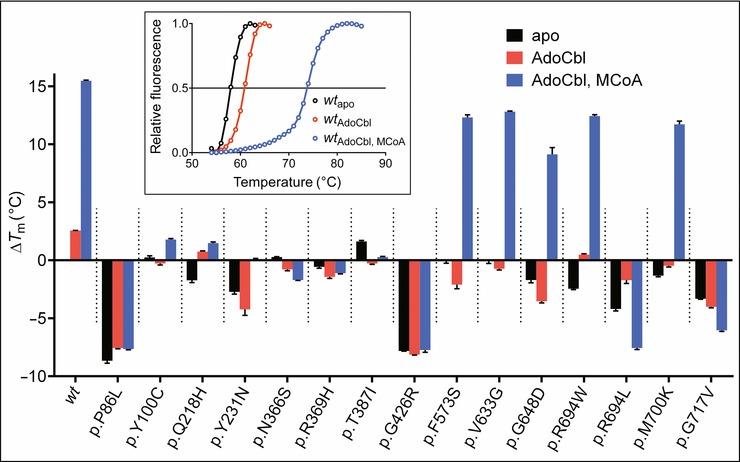
Thermolability of MUT mutations. The change in *T*_m_ values (Δ*T*_m_, compared with *apo wt*) for each mutant MUT in the *apo* state, or following the addition of AdoCbl alone or with AdoCbl and MCoA, is shown. Black, *apo* protein; red, with 50 μM AdoCbl; blue, with 50 μM AdoCbl and 500 μM MCoA. Error bars depict SEM from at least three measurements. (Inset) Representative DSF melting curves of *wt* MUT in the *apo* form (black), with 50 μM AdoCbl (red) and with 50 μM AdoCbl and 500 μM MCoA (blue).

To test whether the catalytic impairment of certain MUT mutants relates to changes in their structural integrity, we expressed and purified those mutants in large scale that were sufficiently soluble in *E. coli* (Fig. 2A, *n* = 15). They had similar retention volumes in size exclusion chromatography as *wt* MUT [Froese et al., 2010b], with no detection of higher order aggregates (data not shown), indicating a native‐like homodimer devoid of global folding defects. To identify any subtle stability changes, we applied a differential scanning fluorimetry (DSF) assay [Vedadi et al., 2006] to follow temperature‐induced protein unfolding in order to measure the melting temperature (*T*_m_) of each purified protein. As proof of principle, we first interrogated the stability effect of physiological ligands (cofactor AdoCbl, substrate analogue malonyl‐CoA) on the *T*_m_ of *wt* MUT, with the rationale that binding of the cognate ligands should stabilize MUT thereby increasing its *T*_m_. Consistent with this, *wt* MUT (at 1 μM) exhibits a *T*_m_ of 58.2°C in the *apo* form, which increases in a dose‐dependent manner upon addition of the native‐like ligands (Supp. Fig. S3A and B), reaching *T*_m_ values of 60.8°C (Δ*T*_m_ = 2.6°C) with AdoCbl (50 μM), and 73.7°C (Δ*T*_m_ = 15.5°C) with both AdoCbl (50 μM) and MCoA (500 μM) (Fig. 3, inset). The extent of these “*T*_m_ shifts” corroborates previous structural observations [Froese et al., 2010b], in which the smaller Δ*T*_m_ by binding AdoCbl alone is consistent with a modest rearrangement in the MUT C‐terminal domain (Supp. Fig. S4A), whereas the larger Δ*T*_m_ with AdoCbl + MCoA is associated with a substantial conformational change in the N‐terminal domain (Supp. Fig. S4B). Notably, MCoA alone (500 μM) did not increase the *T*_m_ of MUT beyond *apo* levels (data not shown), suggesting an ordered binding mechanism of AdoCbl cofactor first, followed by substrate, in the ternary complex.

We subsequently measured the *T*_m_ values of purified mutant proteins in the *apo*, AdoCbl bound, and AdoCbl + MCoA bound states (Fig. 3), to determine if they had a lower *T*_m_ than *wt*, an indicator of protein destabilization [Leandro et al., 2011]. For clarity, the ∆*T*_m_ of *apo* mutants compared to *apo‐wt* is given in Figure 2E. Six of 15 proteins exhibited near‐*wt T*_m_ in their *apo* forms, indicating intact protein integrity. Of these, two (p.F573S, p.V633G) responded to AdoCbl + MCoA stabilization with similar *T*_m_ shifts as *wt*, whereas four (p.Y100C, p.N366S, p.R369H, p.T387I) did not, suggesting that the latter mutants lost the capacity for native ligand stabilization. In particular, p.T387I showed a concentration‐dependent decrease in *T*_m_ when adding AdoCbl alone (Supp. Fig. S3C). The other nine proteins, however, were more thermolabile than *wt* in their *apo* forms. Although five (p.Q218H, p.Y231N, p.G648D, p.R694W, and p.M700K) had slightly lower *T*_m_ than *wt* (Δ*T*_m_ range −2.7 to −1.3) and responded to AdoCbl + MCoA stabilization, four (p.P86L, p.G426R, p.R694L, and p.G717V) were substantially more thermolabile than *wt* (maximum destabilization: p.P86L, Δ*T*_m_ −8.7°C) and did not respond to ligand supplementation.

### Stabilization of MUT Mutants by Osmolytes

**Figure 4:**
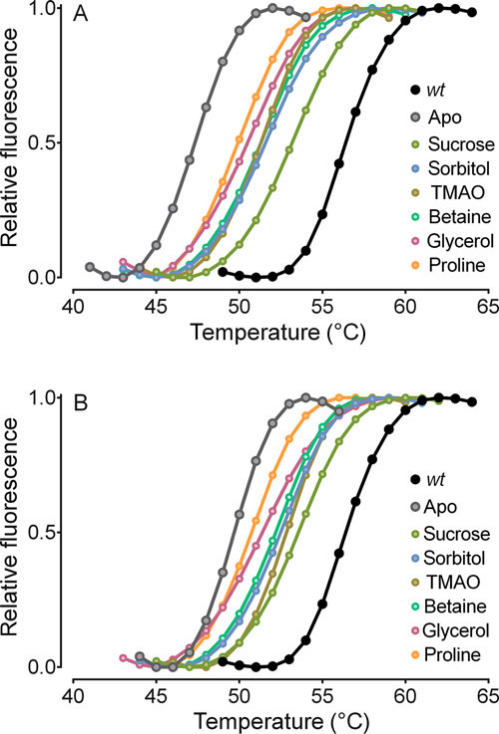
Stabilization of mutant MUT by osmolytes. Representative DSF melting curves for p.P86L (**A**) and p.G426R (**B**) mutants in the *apo* form (gray) and in the presence of osmolytes (colors) are shown along with *wt* MUT (black).

The insolubility and thermolability of a number of MUT mutants, compared with *wt*, implicate protein destabilization as a biochemical defect and prompted us to investigate the potential for posttranslational stabilization of mutant proteins by six different osmolytes using DSF and bacterial expression (Fig. 4; Supp. Figs. S5 and S6). When applied in high millimolar concentrations, these uncharged, low‐molecular‐weight compounds function as “chemical chaperones” and stabilize a number of disease‐associated proteins prone to misfolding, an effect likely mediated by osmotic changes in the milieu [Nascimento et al., 2008; Majtan et al., 2010]. The two most thermolabile MUT mutants in our study, p.P86L and p.G426R, showed that *T*_m_ increases at high osmolyte concentrations (10% glycerol, 600 mM others), with the efficacy of stabilization dependent on the osmolyte used (Fig. 4A and B). These osmolytes confer a nonsaturating dose response, and the effect appears nonselective, as *wt* and all mutant proteins tested demonstrate stabilization in their *apo* forms to similar degrees (Supp. Fig. S5). To determine if osmolytes could also stabilize proteins cotranslationally, we expressed several mutants with varying levels of solubility in *E. coli* and supplemented their growth (Supp. Fig. S6). Compared with no supplement control, soluble protein yield was improved with glycerol (at 1% concentration: p.A191E and p.G426R; 5%: p.R694W), TMAO (100 mM: p.P615T and p.R694W), and to a lesser extent betaine (100 mM: p.R694W). No improvement for p.L328F was seen, whereas *wt* protein was very soluble under all conditions, suggesting the rescue could be mutant specific. Together, our data demonstrate the amenability of small exogenous molecules to modulate MUT stability, and support the notion to develop target‐specific “pharmacological chaperones” (PCs).

## Discussion

Our study aimed to establish a genotype–phenotype correlation by characterizing the biochemistry associated with a set of *MUT* missense mutations. Earlier small‐scale studies have tested the activity and expression of MUT missense mutants, for example, using the lower eukaryote *Saccharomyces cerevisiae* (five mutations) (Crane and Ledley, 1994) and *E. coli* (four mutations) [Janata et al., 1997] as recombinant hosts. Our selected set of 23 mutants, five of which overlap with those previous studies (p.Y231N, p.R369H, p.G648D, p.R694W, p.G717V), constitutes to our knowledge the largest biochemical analysis of MUT mutations to date.

### A Catalogue of Biochemical Defects in MUT Mutants

Traditionally, MMA phenotypes are broadly classified into *mut* ^0^ and *mut* ^−^ subtypes based on the response of a patient cell line to hydroxocobalamin in propionate incorporation [Lempp et al., 2007]. In order to understand the molecular mechanism(s) governing the *mut* ^0^/*mut* ^−^ subtypes and catalogue the associated biochemical defects, we have further assessed MUT mutations based on stability and catalytic properties as summarized in Table 1B.

We evaluated mutants on two stability criteria, *folding* and *thermolability*. *Folding* defects refer to the aggregation or degradation of translated polypeptides, thereby reducing the steady‐state protein. These mutants displayed drastically reduced soluble protein yield, as exemplified by p.P615T/L. Four other mutants, two from each *mut* subtype (*mut* ^0^: p.A191E, p.L328F; *mut* ^−^: p.S344F, p.F573S), also fit this category having reduced detectable protein in one of the two expression systems. A few mutants displayed only slightly decreased soluble *E. coli* expression, such as p.G717V and p.R694W, consistent with a previous report [Crane and Ledley, 1994] and were not included in the *folding* category. *Thermolabile* mutants had near‐*wt* levels of recombinant expression and solubility, reflecting near‐native folding, but increased temperature sensitivity. Four mutations in this study fell into the *thermolabile* category (p.P86L, p.G426R, p.R694L, p.G717V).

Inspection of the MUT crystal structure shows that both *folding* and *thermolabile* mutants involve amino acid changes that (1) remove a conformationally restricted proline residue (e.g., p.P86L and p.P615L/T), (2) cause steric clashes with a bulky side chain (e.g., p.L328F and p.S344F), or (3) disrupt the natively charged/polar (e.g., p.R694L) or nonpolar (e.g., p.A191E) environment. Furthermore, stability mutations are distributed across the entire polypeptide, indicating that all domains of the protein contribute to its intrinsic stability.

In addition to stability mutants, we also identified direct kinetic impairment, which we termed *catalytic* and *K*_M_ mutants. *Catalytic* mutants had low residual activity (<5% of *wt*) despite detectable eukaryotic expression and constituted the largest category of mutants (10 in this study, Table 1B). Of particular note, two *catalytic* mutants, p.Q218H and p.A219E, involve structurally adjacent residues lining the substrate‐binding channel (Fig. 1A; Supp. Fig. S1D and E), where p.Q218 has been implicated in transition state stabilization [Loferer et al., 2003]. *K*_M_ mutants exhibited >200‐fold increased *K*_M_ for AdoCbl compared with *wt*. Among the seven *K*_M_ mutants identified (Table 1B), only three (p.V633G, p.G648D, and p.G717V) are buried within the C‐terminal cobalamin‐binding domain (Fig. 1A; Supp. Fig. S1P, Q, and T). The other four are toward the N‐terminal end: p.Y231N is situated at the interdomain interface, within a loop that contacts the cobalamin corrin ring (Supp. Fig. S1F); p.G426R is situated at the dimeric interface; and p.P86L and p.Y100C are located near the N‐terminal substrate‐binding channel, distant from any obvious involvement in cobalamin binding. Further investigation is therefore warranted to understand how the *K*_M_ effect of these mutations is mediated.

It is of note that our classification scheme is not aimed at *pigeonholing* mutant alleles into disparate, mutually exclusive categories; on the contrary, there is a high degree of interdependence between various stability and catalytic properties that together contribute to proper enzyme function. This is clearly exemplified with seven mutants fitting multiple criteria. Three of them had a significantly increased *K*_M_ but were also thermolabile (p.P86L, p.G426R, and p.G717V), one was *thermolabile* with a *catalytic* dysfunction (p.R694L), and three met the criteria of *folding* and *catalytic* defects (p.A191E, p.L328F, p.S344F). In addition, three mutations were termed *unclear* (p.Y316C, p.T387I, and p.L736F) because in our assays they lack definitive aberration in stability and enzyme kinetics, showed detectable protein by Western blot, intermediate enzyme activity, and only moderate *K*_M_ change.

Future studies should aim to address the lesser characterized biochemical aspects of MUT and possible mutational effects, which are beyond the scope of this study. These include (1) possible allostery between substrate and cofactor binding, which could potentially explain the *T*_m_ shift from MCoA + AdoCbl but not MCoA alone; (2) potential formation of a supramolecular complex with the GTPase MMAA and adenosyltransferase MMAB [Froese and Gravel, 2010], required for the proper assembly of AdoCbl onto the MUT enzyme; and (3) interallelic rescue of certain MUT disease alleles (e.g., p.R93H and p.G717V) [Worgan et al., 2006].

### Clinical Utility and Implications of In Vitro Characterization

A clinical application for our biochemical categorization of *MUT* genotypes is to help prognosticate the course of the disease. As expected, the characterized *mut* ^0^ mutants harbor *folding* or *catalytic* defects, resulting in very low or undetectable residual enzyme activities consistent with their predominance in neonatal onset patients with severe long‐term complications. On the contrary, the characterized *mut* ^−^ mutants can present in any one, or combinations, of the four biochemical defects defined here. We postulate that mutations that do not interfere with protein stability and have less of a catalytic penalty would result in milder disease (e.g., p.P86L or p.G426R). Similar to other metabolic diseases where an activity threshold of ∼5%–10% of *wt* is sufficient to ameliorate disease [Leinekugel et al., 1992; Suzuki, 2013], *mut* ^−^ patients often show a later onset and less long‐term complications, presumably due to the intermediate level of residual MUT activity.

Whether any MUT‐deficient patient, including those of the *mut^−^* subtype, benefit from treatment with high doses of hydroxocobalamin is controversial. Early publications documented no response [Wilcken et al., 1977; Matsui et al., 1983] and a recent documentation of a possible response has relied on retrospective reviews [Horster et al., 2007], whereas up to now, no controlled studies clearly demonstrating response to hydroxocobalamin have been published. This picture is very different from PKU, where >40% patients respond clinically to the cognate cofactor tetrahydropterin (BH_4_) of phenylalanine hydroxylase (PAH) [Zurfluh et al., 2008]. For *mut‐*type MMA, a clinical response to treatment is usually defined as reduced plasma or urinary concentration of methylmalonic acid. Although difficult to measure because of its broad variation in body fluids [Thompson and Chalmers, 1990; Horster et al., 2007; Fowler et al., 2008], and clinical improvement even more difficult to determine reliably, lower methylmalonic acid levels are thought to reflect a higher residual enzyme activity protecting patients from severe long‐term complications (e.g., chronic renal failure and neurological complications) [Thompson and Chalmers, 1990; Horster et al., 2007; Cosson et al., 2009]. Therefore, careful evaluation of potential responsiveness to hydroxocobalamin treatment in all MMA patients is warranted, and a protocol has been proposed [Fowler et al., 2008]. Our thermal denaturation data offer a molecular explanation to a possible mechanism of cobalamin treatment by revealing AdoCbl‐induced stability improvement in certain mutants in a ligand‐specific manner similar to that of BH_4_ on mutant PAH. Our results also predict that patients harboring a *K*_M_ defect (e.g., p.G648D) are more likely to benefit from cobalamin treatment based on the assumption that increased hydroxocobalamin plasma levels result in increased cellular AdoCbl production because, in our enzyme assay, these mutants exhibited high activity levels in the presence of high AdoCbl concentration. Nevertheless, hydroxocobalamin treatment may only benefit a small subset of patients with intermediate to high residual activity, if any, outlining an imperative to search for alternative therapies.

### Small Molecule Chaperones as Alternative Therapy?

Our stability data add MUT to the growing list of metabolic enzymes in which mutation‐induced protein destabilization is widely believed to play a causative role in disease pathogenesis [Gregersen et al., 2006]. In this model, mutant polypeptides tend to misfold, aggregate, and be degraded by protein quality control in the cell. Chemical or pharmacologic approaches to partially correct misfolding, divert the mutant polypeptide from degradation/aggregation pathways, and deliver it to the native subcellular destination may therefore allow a sufficient recovery of physiological function. This small molecule “chaperoning” approach has been proposed as a viable treatment for a number of protein misfolding diseases [Pey et al., 2008; Gomes, 2012]. We provide proof of principle for MUT chaperone therapy by showing in vitro and in cell stabilization of several mutant proteins using chemical osmolytes. These small organic solutes enhance protein stability via a nonselective, nontarget‐specific mode of preferential exclusion from the protein's surface [Arakawa and Timasheff, 1985], although necessitating high effective concentrations (in millimolar range) that may be toxic to the cell—restricting their therapeutic utility.

Alternatively, target‐specific PCs could be developed for MUT that, unlike chemical chaperones, exert a direct stabilization effect by binding to the affected protein, potentially allowing lower, clinically tolerable dosage concentrations. PCs have shown promise in several lysosomal storage diseases, entering early‐stage clinical trials for Gaucher, Tay‐Sachs, and Fabry diseases [Boyd et al., 2013]. In the case of MUT, we anticipate that an effective PC molecule need only to stabilize the mutant protein “long enough” to be delivered to mitochondria, where it can be further stabilized by its native ligands and protein interaction partners (MMAA and MMAB). Such a PC molecule may increase the residual enzyme activity of the mutant protein to a sufficient level that copes with the cellular demands of propionate catabolism. Therefore, PC therapy is likely to rescue mild stability defects (i.e., alleles that give rise to destabilized protein and partial loss of function; e.g., p.G426R, p.M700K, and p.G717V), either to be administered alone or as a complement to the existing diet/hydroxocobalamin treatment. Other *catalytic*, *K*_M_ mutations, without stability defects, may also benefit from PCs that potentially increase residual activity, acting as “enzymatic activators.” Altogether, the stage is set for a systematic, nonbiased screening regime in the search for PC molecules targeting MUT, and our established DSF assay may provide a good starting point for this screening.

## Supporting information

Disclaimer: Supplementary materials have been peer‐reviewed but not copyedited.

**Figure S1**. Structural view of the amino acid environment for each mutation in this study. For each panel, the amino acid of interest is coloured cyan. Secondary structure elements are coloured cyan for the N‐terminal substrate binding domain, yellow for inter‐domain linker, and magenta for C‐terminal cobalamin binding domain. For panels **a, b, d, e, i** and **l**, malonyl‐CoA is shown as sticks (yellow carbon atoms). For panels **d, k, p, q, s** and **t**, adenosylcobalamin is shown as sticks (black carbon atoms). For panels **a, g, k** and **m**, the neighboring subunit in the MUT dimer is shown as black cartoon. For panels **c, h, m, o** and **q**, amino acids surrounding the site of interest are also shown in spheres, to highlight the tight steric packing. Where applicable, hydrogen bonds are shown as dashed lines (distance in angstrom). An interactive ver‐sion of this structural representation is available at www.thesgc.org/MUT.
**Figure S2**. Enzyme activities of MUT *wt* and mutants in decreasing order. Each bar represents the mean of at least two replicate experiments (error bars depict SEM). Black bars indicate *high* (50‐100% of *wt*, n=5), dotted bars *intermediate* (6‐49% of *wt*, *n*=5) and white bars *low* (0‐5% of *wt*, *n*=13) levels of ac‐tivity
**Figure S3**. Ligand‐dependent thermal denaturation of MUT. *wt* MUT is stabilized with increasing concentrations of AdoCbl (**A**) and malonyl‐CoA (**B**). Mutant p.T387I is destabilized with increasing AdoCbl concentrations (**C**).
**Figure S4**. Substrate/cofactor‐induced conformational changes in MUT. **A**. Superposition of MUT structures in the *apo* and AdoCbl‐bound (*holo*) states shows modest rearrangement in the C‐terminal domain (boxed) by the binding of AdoCbl alone. **B**. Superposition of MUT structures in the *holo* and ternary (AdoCbl and MCoA bound) states reveals substantial conformational changes in the N‐terminal domain (boxed) by the additional binding of MCoA. Ligands are shown in sticks (AdoCbl, yellow carbon; MCoA, green carbon. (PDB codes: *apo*, 2XIQ; *holo*, 2XIJ; ternary, 2XIQ).
**Figure S5**. Concentrations‐dependent thermal stabilization in wild‐type and mutant MUT upon addition of chemical chaperones: **A**. proline, **B**. glycerol, **C**. betaine, **D**. sorbitol, **E**. TMAO, **F**. sucrose.
**Figure S6**. SDS‐PAGE of test purification for wild‐type, p.A191E, p.L328F, p.G426R, p.P615T and p.R694W after co‐translational expression in the presence of chemical osmolytes. Glycerol (at 1% and 5%), TMAO and betaine at 100 mM were tested. For clarity, only the relevant gel section is shown. CC: chemical chaperone; TLC: total cell lysate; E: eluant after affinity purification; marker on left: sizes in kDa.
**Table S1**. Primers used for site‐directed mutagenesis of pTracer‐MUT *wt* and pNIC‐MUT *wt* constructs
**Table S2**. Methylmalonyl‐CoA mutase (MUT) activities and KM values for the cofactor, ade‐nosylcobalamin (AdoCbl)
