# Supplementary material for: Functional Characterization and Categorization of Missense Mutations that Cause Methylmalonyl‐CoA Mutase (MUT) Deficiency
Source: Hum Mutat. 2014 Nov 24;35(12):1449–58. doi: 10.1002/humu.22633 (PMC4441004; doi:10.1002/humu.22633)
Supplement: Supplementary file 1 — Figure S1. Structural view of the amino acid environment for each mutation in this study. For each panel, the amino acid of interest is coloured cyan. Secondary structure elements are coloured cyan for the N‐terminal substrate binding domain, yellow for inter‐domain linker, and magenta for C‐terminal cobalamin binding domain. For panels a, b, d, e, i and l, malonyl‐CoA is shown as sticks (yellow carbon atoms). For panels d, k, p, q, s and t, adenosylcobalamin is shown as sticks (black carbon atoms). For panels a, g, k and m, the neighboring subunit in the MUT dimer is shown as black cartoon. For panels c, h, m, o and q, amino acids surrounding the site of interest are also shown in spheres, to highlight the tight steric packing. Where applicable, hydrogen bonds are shown as dashed lines (distance in angstrom). An interactive ver‐sion of this structural representation is available at www.thesgc.org/MUT. Figure S2. Enzyme activities of MUT wt and mutants in decreasing order. Each bar represents the mean of at least two replicate experiments (error bars depict SEM). Black bars indicate high (50‐100% of wt, n=5), dotted bars intermediate (6‐49% of wt, n=5) and white bars low (0‐5% of wt, n=13) levels of ac‐tivity Figure S3. Ligand‐dependent thermal denaturation of MUT. wt MUT is stabilized with increasing concentrations of AdoCbl (A) and malonyl‐CoA (B). Mutant p.T387I is destabilized with increasing AdoCbl concentrations (C). Figure S4. Substrate/cofactor‐induced conformational changes in MUT. A. Superposition of MUT structures in the apo and AdoCbl‐bound (holo) states shows modest rearrangement in the C‐terminal domain (boxed) by the binding of AdoCbl alone. B. Superposition of MUT structures in the holo and ternary (AdoCbl and MCoA bound) states reveals substantial conformational changes in the N‐terminal domain (boxed) by the additional binding of MCoA. Ligands are shown in sticks (AdoCbl, yellow carbon; MCoA, green carbon. (PDB codes: apo, 2XIQ; holo, 2XIJ; ternary [file HUMU-35-1449-s001.pdf]

Functional characterization and categorization of missense mutations that cause methylmalonyl-CoA mutase deficiency

Supporting Information

Patrick Forny[1,2,ç], D. Sean Froese[1,3,ç], Terttu Suormala[1], Wyatt W. Yue[3,#], Matthias R. Baumgartner[1,2,#]

[1]Division for Metabolic Disorders and Children's Research Center, University Children's Hospital, Zurich, Switzerland CH-8032

[2]Zurich Center for Integrative Human Physiology, University of Zurich, Switzerland

[3]Structural Genomics Consortium, University of Oxford, UK OX3 7DQ

[ç]Patrick Forny and D. Sean Froese contributed equally to this work.

[#]To whom correspondence may be addressed:

Matthias R. Baumgartner

Tel: +41 (0)44 266 7722

Fax: +41 (0)44 266 7167

Email: matthias.baumgartner@kispi.uzh.ch

Wyatt W. Yue

Tel: +44 (0)1865 617757

Fax: +44 (0)1865 617575

Email: wyatt.yue@sgc.ox.ac.uk

Supp. Figure S1

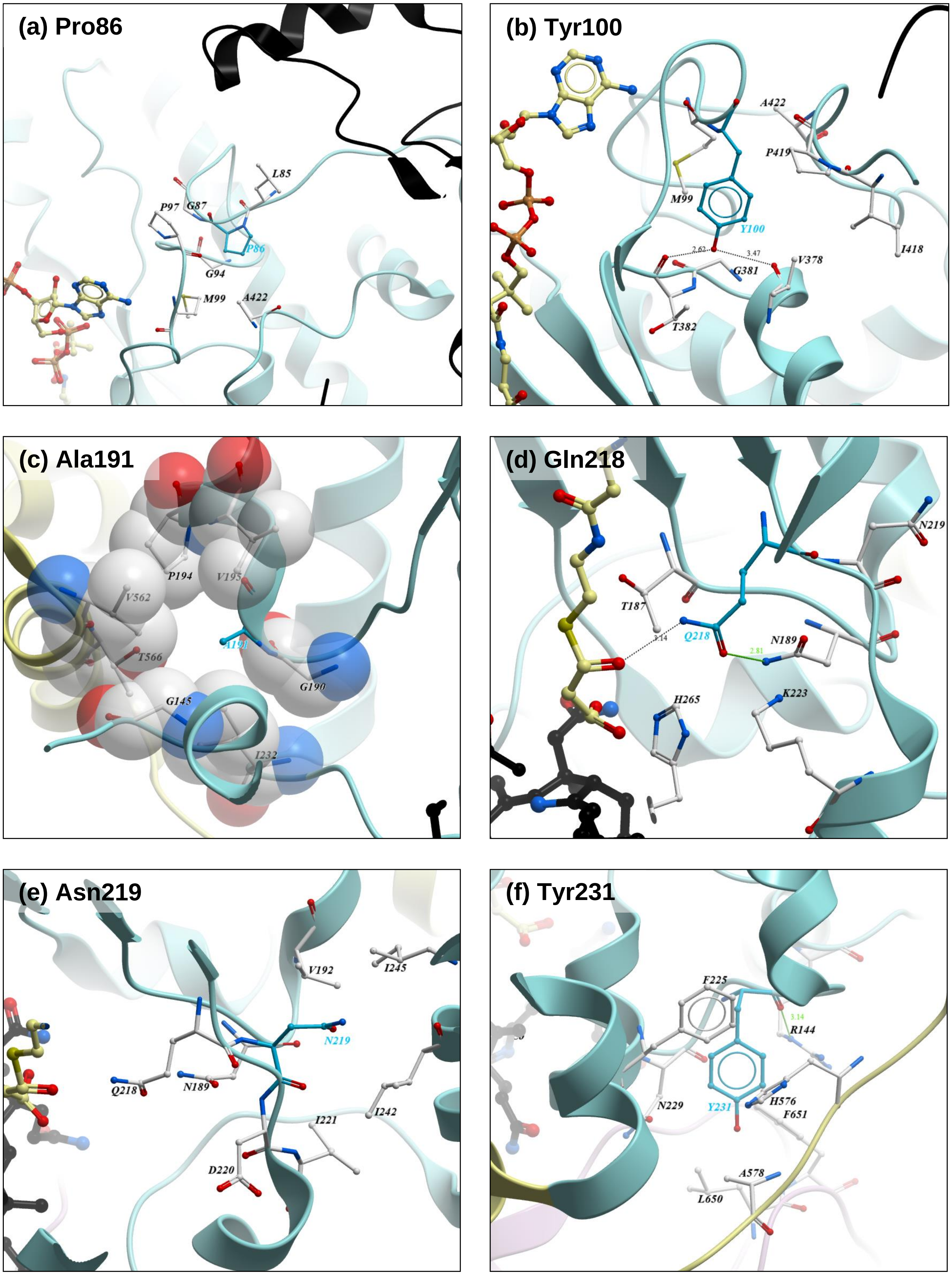

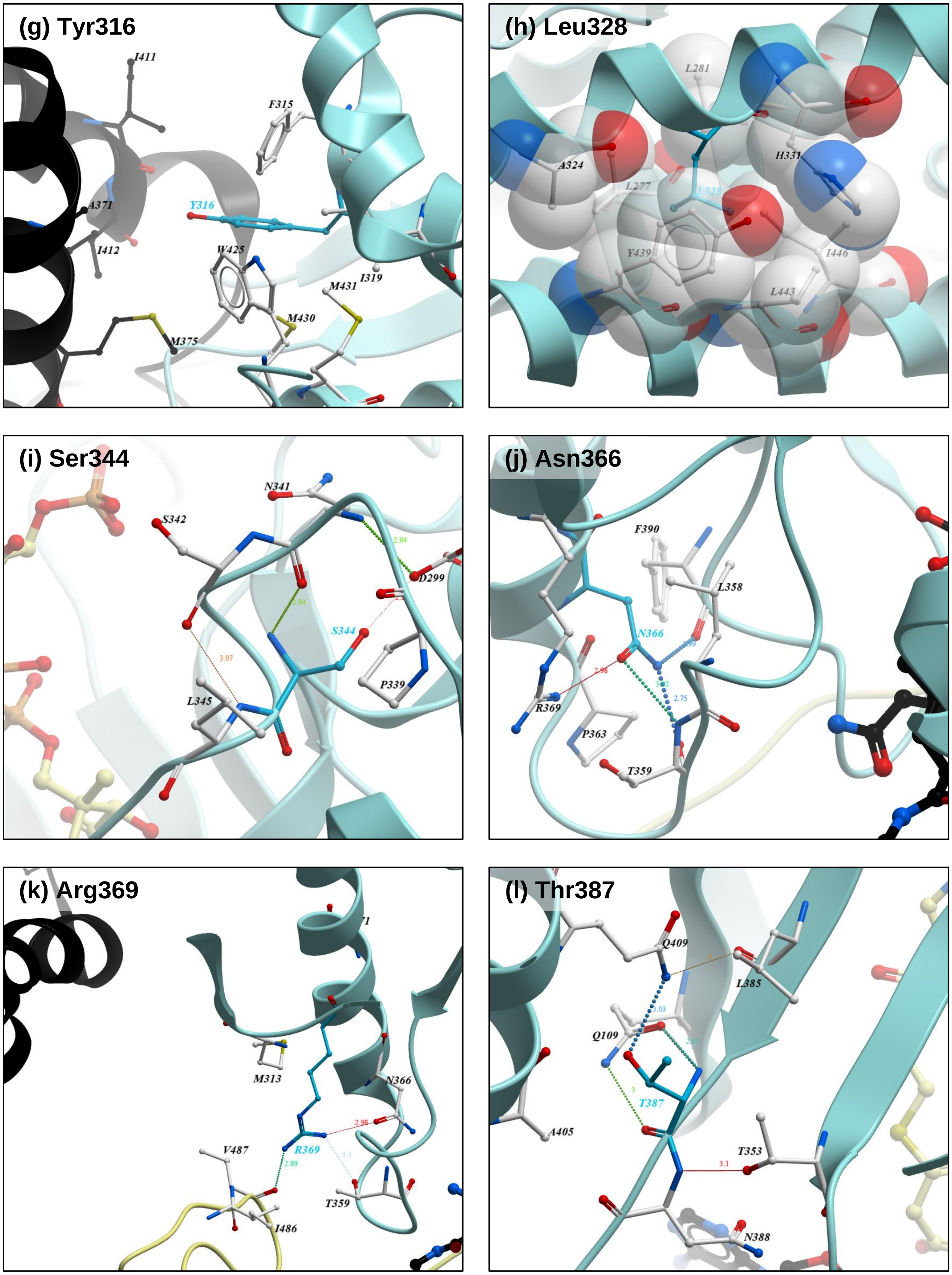
(g) Tyr316
I411
F315
Y316
A371
I412
W425
M431
I319
M430
M375
(h) Leu328
L281
A324
H331
L277
L328
Y439
I446
L443
(i) Ser344
N341
S342
D299
S344
3.07
P339
L345
(j) Asn366
F390
L358
N366
2.98
R369
P363
2.75
T359
(k) Arg369
M313
N366
2.98
V487
R369
2.89
T359
I486
(l) Thr387
Q409
L385
Q109
T387
A405
T353
3.1
N388

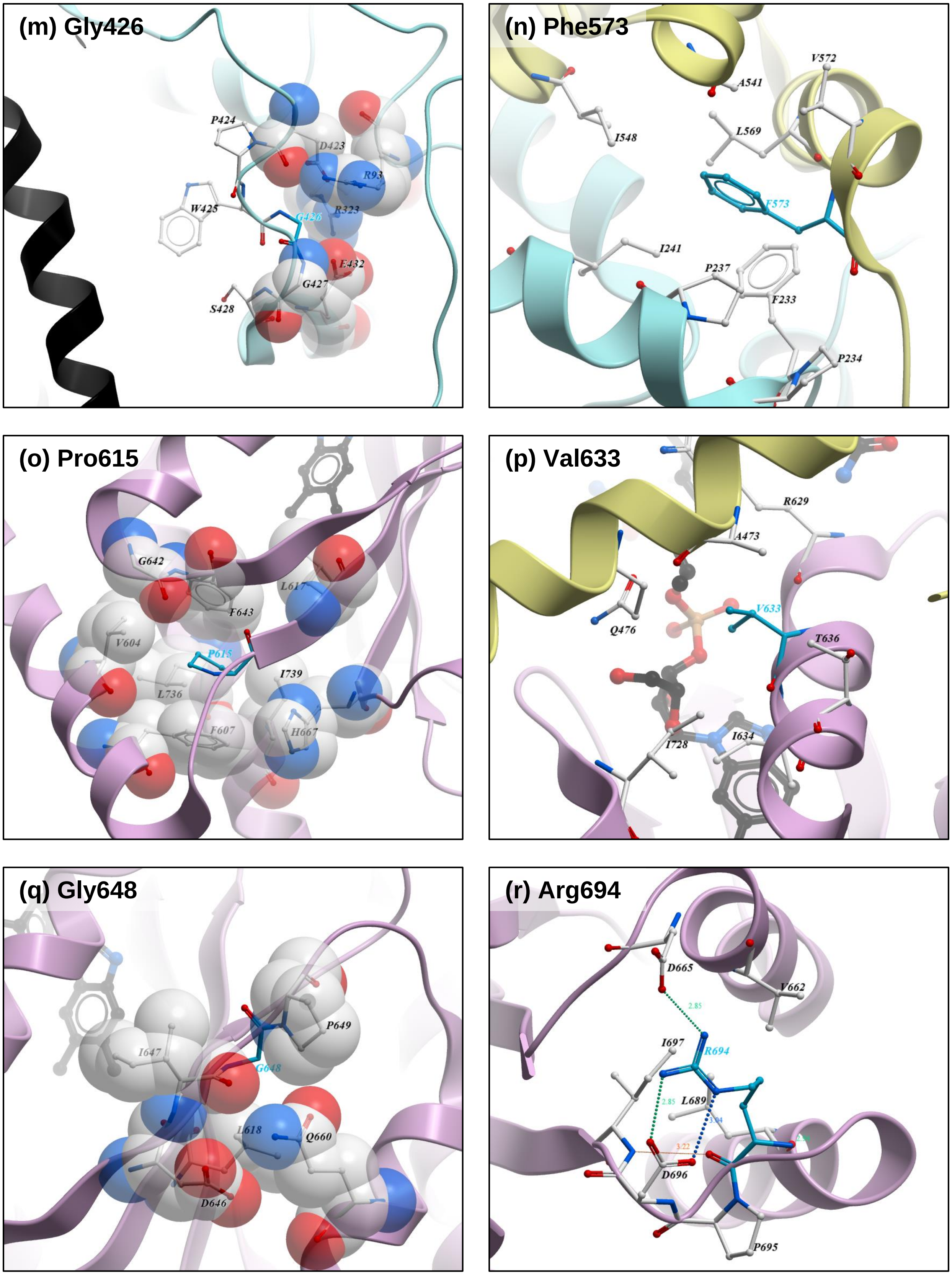

(m) Gly426
P424
D423
R93
W425
G426
R323
E432
G427
S428
(n) Phe573
V572
A541
I548
L569
F573
I241
P237
F233
P234
(o) Pro615
G642
L617
F643
V604
P615
I739
L736
F607
H667
(p) Val633
R629
A473
Q476
V633
T636
I634
I728
(q) Gly648
P649
I647
G648
L618
Q660
D646
(r) Arg694
D665
V662
2.85
I697
R694
L689
D696
P695

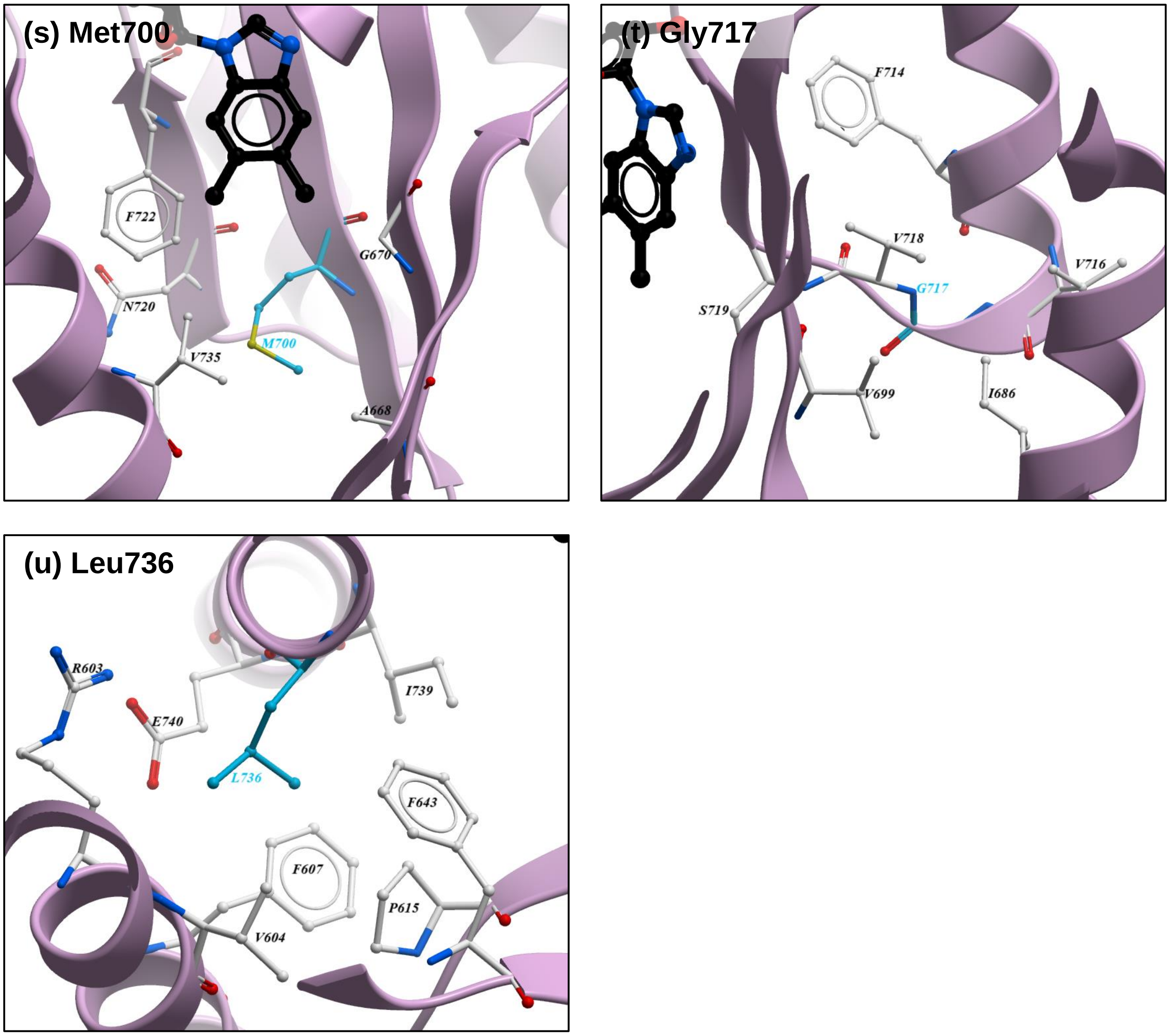

Supp. Figure S1. Structural view of the amino acid environment for each mutation in this study. For each panel, the amino acid of interest is coloured cyan. Secondary structure elements are coloured cyan for the N-terminal substrate binding domain, yellow for inter-domain linker, and magenta for C-terminal cobalamin binding domain. For panels **a, b, d, e, i** and **l**, malonyl-CoA is shown as sticks (yellow carbon atoms). For panels **d, k, p, q, s** and **t**, adenosylcobalamin is shown as sticks (black carbon atoms). For panels **a, g, k** and **m**, the neighboring subunit in the MUT dimer is shown as black cartoon. For panels **c, h, m, o** and **q**, amino acids surrounding the site of interest are also shown in spheres, to highlight the tight steric packing. Where applicable, hydrogen bonds are shown as dashed lines (distance in angstrom). An interactive version of this structural representation is available at *www.thesgc.org/MUT*.

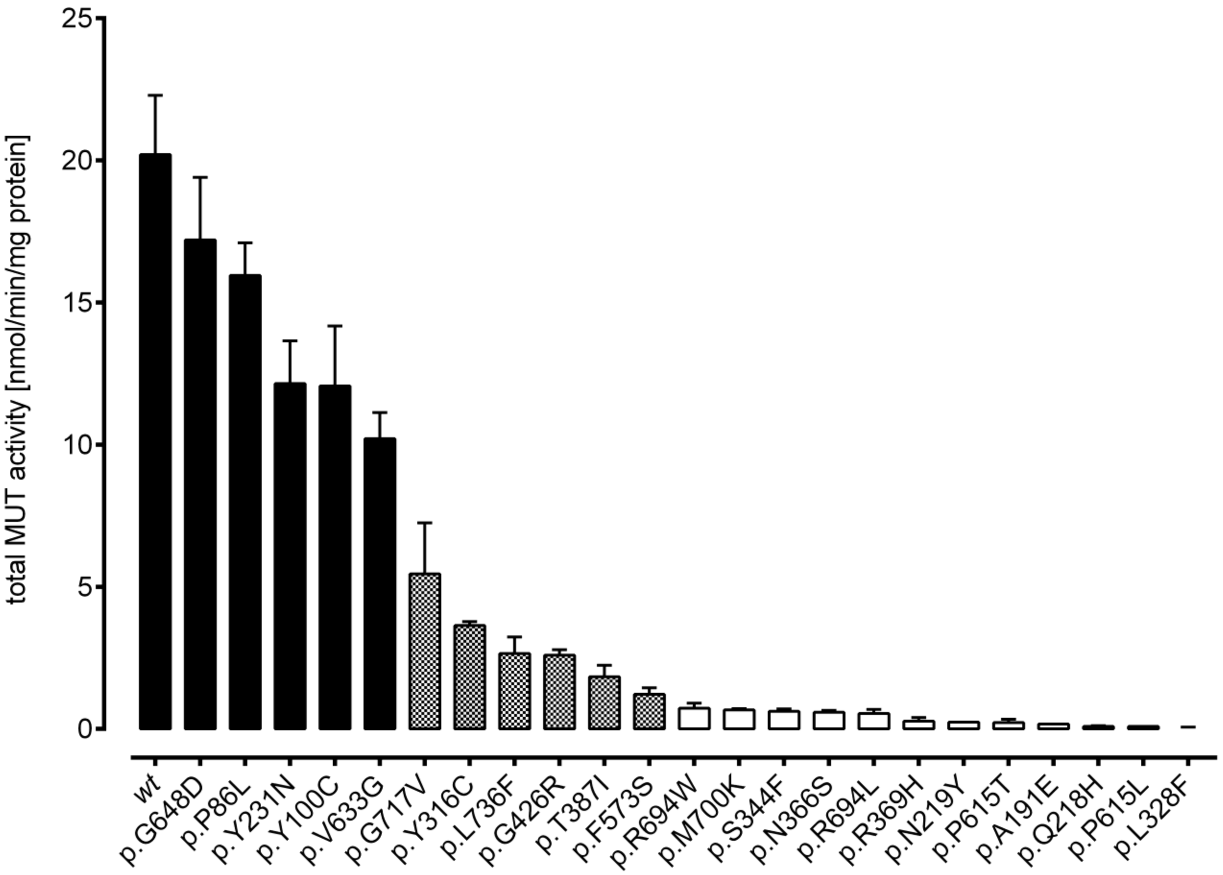

Supp. Figure S2. Enzyme activities of MUT *wt* and mutants in decreasing order. Each bar represents the mean of at least two replicate experiments (error bars depict SEM). Black bars indicate *high* (50-100% of *wt*, n=5), dotted bars *intermediate* (6-49% of *wt*, *n*=5) and white bars *low* (0-5% of *wt*, *n*=13) levels of activity.

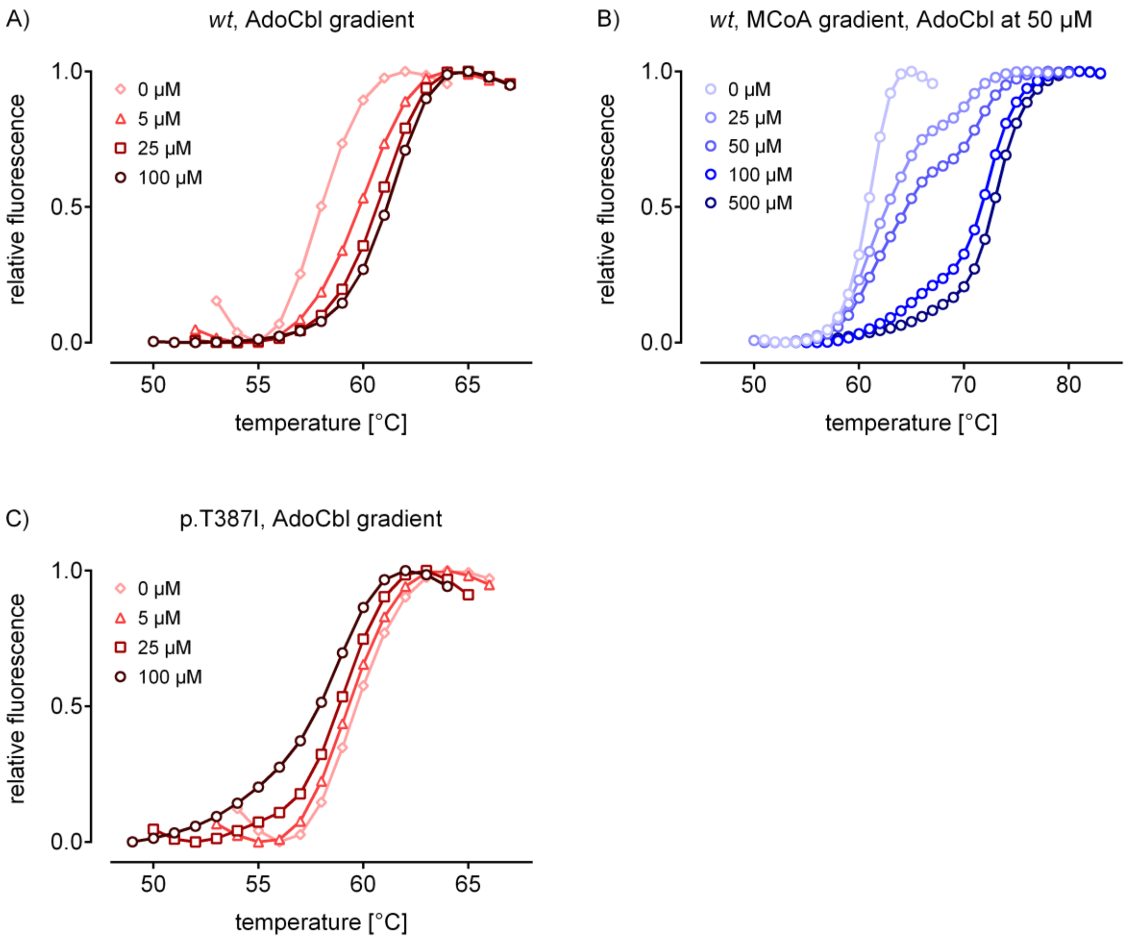

Supp. Figure S3. Ligand-dependent thermal denaturation of MUT. *wt* MUT is stabilized with increasing concentrations of AdoCbl (**A**) and malonyl-CoA (**B**). Mutant p.T387I is destabilized with increasing AdoCbl concentrations (**C**).

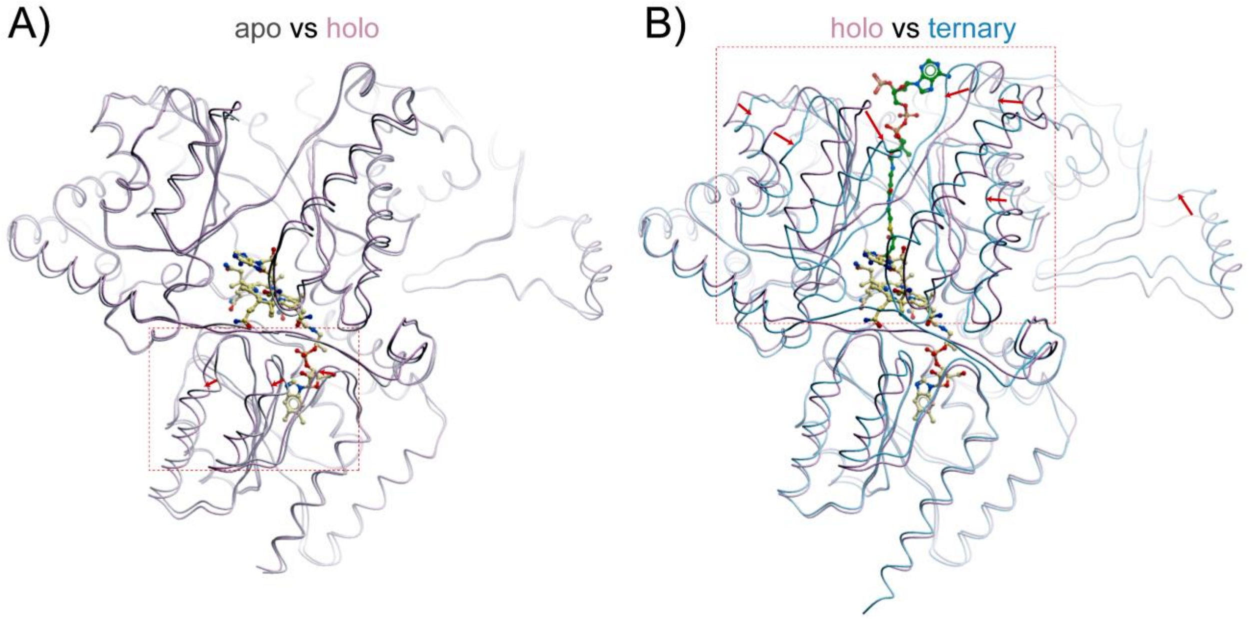

Supp. Figure S4. Substrate/cofactor-induced conformational changes in MUT. **A.** Superposition of MUT structures in the *apo* and AdoCbl-bound (*holo*) states shows modest rearrangement in the C-terminal domain (boxed) by the binding of AdoCbl alone. **B.** Superposition of MUT structures in the *holo* and ternary (AdoCbl and MCoA bound) states reveals substantial conformational changes in the N-terminal domain (boxed) by the additional binding of MCoA. Ligands are shown in sticks (AdoCbl, yellow carbon; MCoA, green carbon. (PDB codes: *apo*, 2XIQ; *holo*, 2XIJ; ternary, 2XIQ).

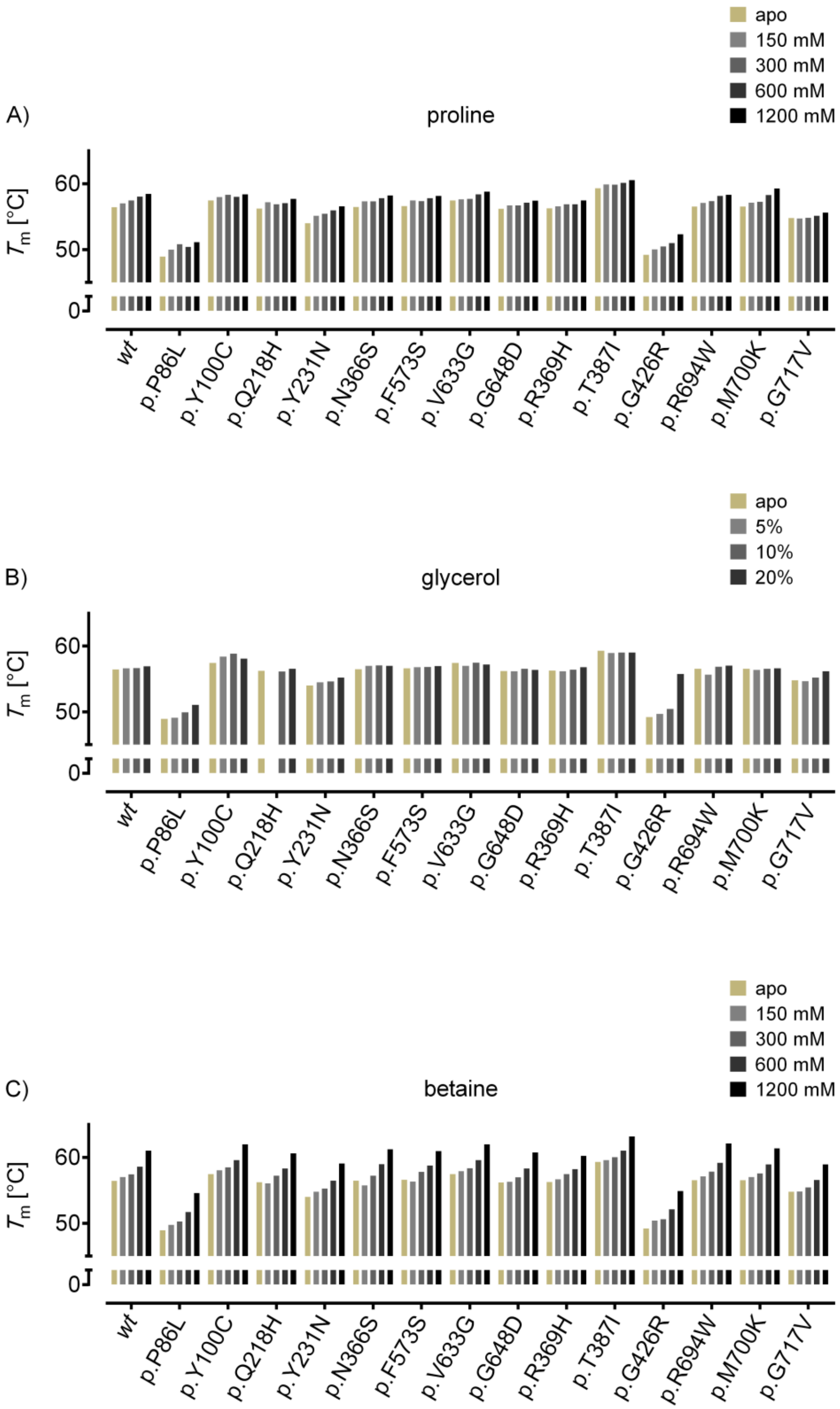
A)
proline
apo
150 mM
300 mM
600 mM
1200 mM
T_m [°C]
60
50
0
wt
p.P86L
p.Y100C
p.Q218H
p.Y231N
p.N366S
p.F573S
p.V633G
p.G648D
p.R369H
p.T387I
p.G426R
p.R694W
p.M700K
p.G717V
B)
glycerol
apo
5%
10%
20%
C)
betaine
apo
150 mM
300 mM
600 mM
1200 mM

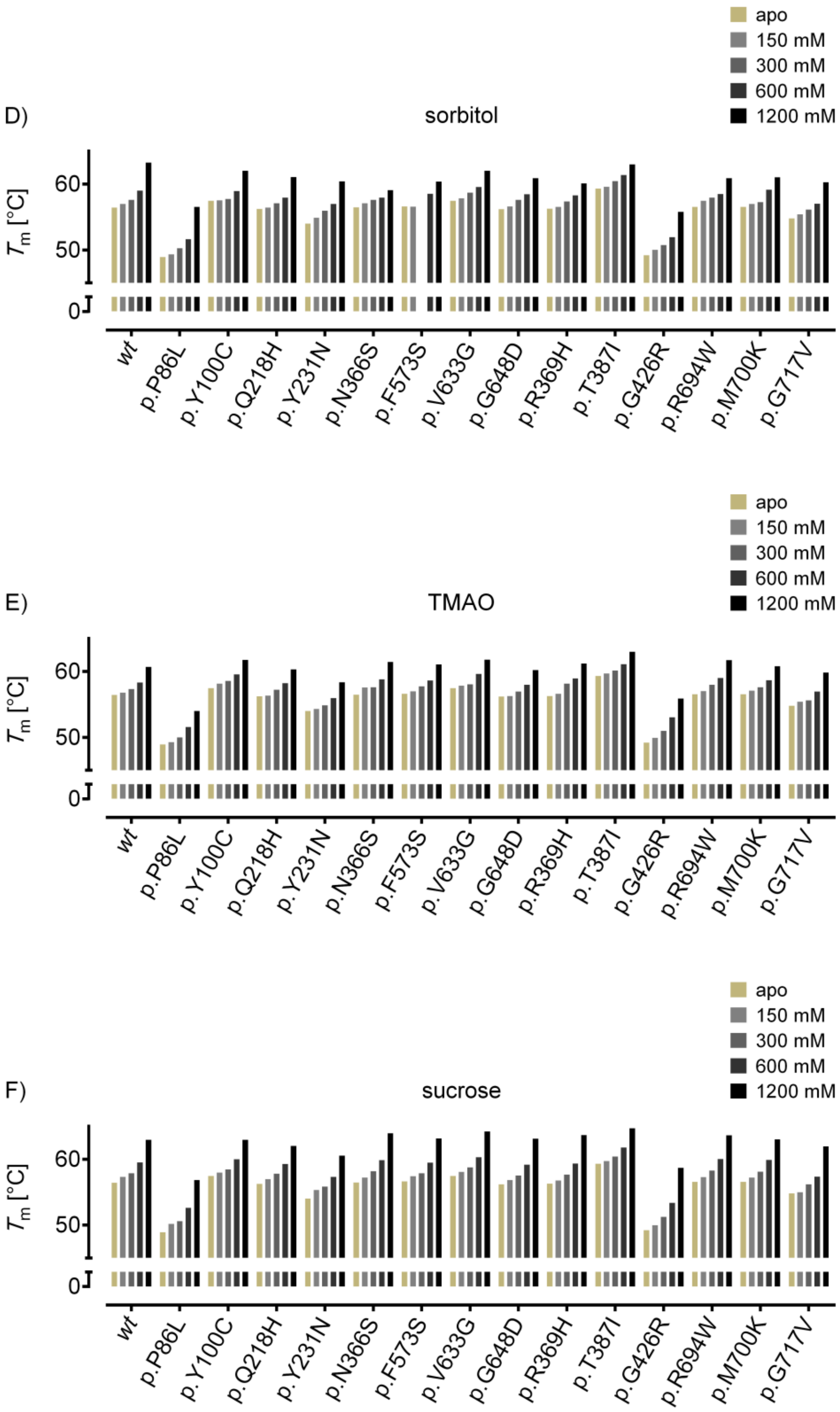

Supp. Figure S5. Concentrations-dependent thermal stabilization in wild-type and mutant MUT upon addition of chemical chaperones: **A.** proline, **B.** glycerol, **C.** betaine, **D.** sorbitol, **E.** TMAO, **F.** sucrose.

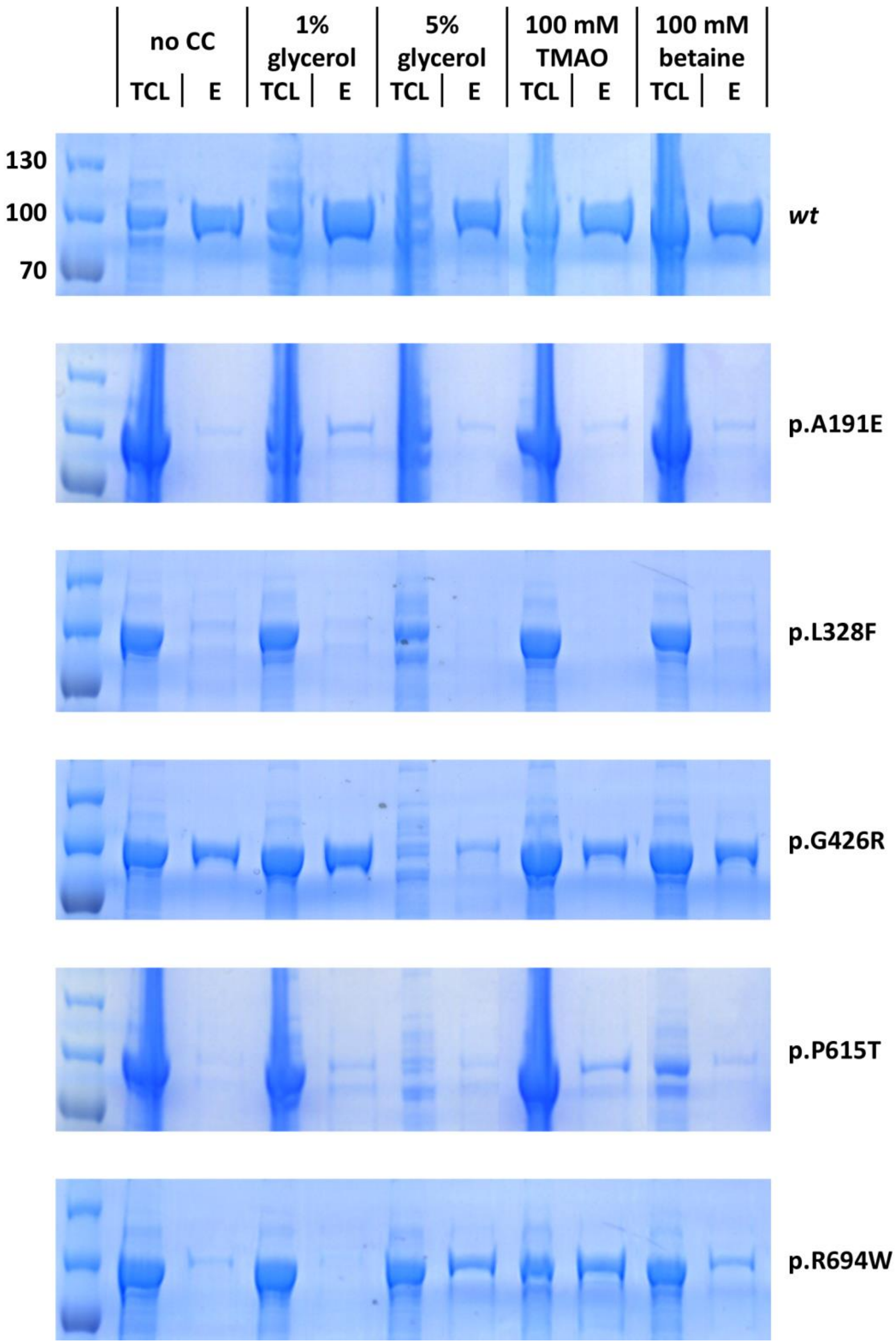

Supp. Figure S6. SDS-PAGE of test purification for wild-type, p.A191E, p.L328F, p.G426R, p.P615T and p.R694W after co-translational expression in the presence of chemical osmolytes. Glycerol (at 1% and 5%), TMAO and betaine at 100 mM were tested. For clarity, only the relevant gel section is shown. CC: chemical chaperone; TLC: total cell lysate; E: eluant after affinity purification; marker on left: sizes in kDa.

Supp. Table S1. Primers used for site-directed mutagenesis of pTracer-MUT *wt* and pNIC-MUT *wt* constructs

amino acid change	F/R	primer sequence 5'-3'
p.P86L	Forward	GACTTACCTGAAGAACTTCTAGGAGTGAAGCCA
p.P86L	Reverse	TGTGAATGGCTTCACTCCTAGAAGTTCTTCAGG
p.Y100C	Forward	GGACCATATCCTACCATGTGTACCTTTAGGCC
p.Y100C	Reverse	GGTCCAGGGCCTAAAGGTACACATGGTAGGATA
p.A191E	Forward	TCCATGACTATGAATGGAGAAGTTATTCCAGTT
p.A191E	Reverse	TGCAAGAACTGGAATAACTTCTCCATTCATAGT
p.Q218H	Forward	AAACTTACTGGTACCATCCACAATGATATACTA
p.Q218H	Reverse	TTCCTTTAGTATATCATTGTGGATGGTACCAGT
p.N219Y	Forward	CTTACTGGTACCATCCAATATGATATACTAAAG
p.N219Y	Reverse	AAATTCCTTTAGTATATCATATTGGATGGTACC
p.Y231N	Forward	TTTATGGTTCGAAATACAAACATTTTTCCTCCA
p.Y231N	Reverse	TGGTTCTGGAGGAAAAATGTTTGTATTTCGAAC
p.Y316C	Forward	GGAATTGGAATGAATTTCTGTATGGAAATAGCA
p.Y316C	Reverse	CATCTTTGCTATTTCCATACAGAAATTCATTCC
p.L328F	Forward	ATGAGAGCTGGTAGAAGATTCTGGGCTCACTTA
p.L328F	Reverse	CTCTATTAAGTGAGCCCAGAATCTTCTACCAGC
p.S344F	Forward	CAGCCTAAAAACTCAAAATTTCTTCTTCTAAGA
p.S344F	Reverse	GTGTGCTCTTAGAAGAAGAAATTTTGAGTTTTT
p.N366S	Forward	GAGCAGGATCCCTACAATAGTATTGTCCGTACT
p.N366S	Reverse	TATTGCAGTACGGACAATACTATTGTAGGGATC
p.R369H	Forward	CCCTACAATAATATTGTCCATACTGCAATAGAA
p.R369H	Reverse	CATTGCTTCTATTGCAGTATGGACAATATTATT
p.T387I	Forward	GGGACTCAGTCTTTGCACATAAATTCTTTTGAT
p.T387I	Reverse	AGCTTCATCAAAAGAATTTATGTGCAAAGACTG
p.G426R	Forward	AAAGTGGCTGATCCTTGGAGAGGTTCTTAC
p.G426R	Reverse	CATCATGTAAGAACCTCTCCAAGGATCAGC
p.F573S	Forward	GATGCCCTGAAAAAGGTATCTGGTGAACATAAA
p.F573S	Reverse	ATTCGCTTTATGTTCACCAGATACCTTTTTCAG
p.P615T	Forward	GAACGTGAAGGTCGCAGAACTCGTCTTCTTGTA
p.P615T	Reverse	TTTTGCTACAAGAAGACGAGTTCTGCGACCTTC
p.P615L	Forward	GAACGTGAAGGTCGCAGACTTCGTCTTCTTGTA
p.P615L	Reverse	TTTTGCTACAAGAAGACGAAGTCTGCGACCTTC
p.V633G	Forward	CATGACAGAGGAGCAAAAGGTATTGCTACAGGA
p.V633G	Reverse	AGCAAATCCTGTAGCAATACCTTTTGCTCCTCT
p.G648D	Forward	GGTTTTGATGTGGACATAGACCCTCTTTTCCAG
p.G648D	Reverse	AGGAGTCTGGAAAAGAGGGTCTATGTCCACATC
p.R694W	Forward	GAACTTAACTCCCTTGGATGGCCAGATATT
p.R694W	Reverse	GACAAGAATATCTGGCCATCCAAGGGAGTT
p.R694L	Forward	GAACTTAACTCCCTTGGACTGCCAGATATT
p.R694L	Reverse	GACAAGAATATCTGGCAGTCCAAGGGAGTT
p.M700K	Forward	CGGCCAGATATTCTTGTCAAGTGTGGAGGGGTG
p.M700K	Reverse	TGGTATCACCCCTCCACACTTGACAAGAATATC
p.G717V	Forward	ATTTCTGTTTGAAGTTGTTGTTTCCAATGT
p.G717V	Reverse	CAAATACATTGGAAACAACAACTTCAAACA
p.L736F	Forward	AAGGCTGCCGTTCAGGTGTTTGATGATATTGAG
p.L736F	Reverse	ACACTTCTCAATATCATCAAACACCTGAACGGC

Supp. Table S2. Methylmalonyl-CoA mutase (MUT) activities and K_M values for the cofactor, adenosylcobalamin (AdoCbl)

		MUT activity, nmol/min/mg protein[¶]					K_M for AdoCbl, nM		
		holo-MUT (–AdoCbl)		total MUT (+AdoCbl)					
**amino acid change**	***mut* class**	**single values**	**mean**	**single values**	**mean**	**% of *wt***	**single values**	**mean**	**times *wt***
p.P86L	mut^-	0.03, 0.20	0.12	17.1, 14.8	16.0	79	3014	3014	636
p.Y100C	mut^-	0.02, 0.05, 0.07	0.05	8.49, 11.9, 15.8	12.1	60	3648	3648	770
p.A191E	mut^0	0.21	0.21	0.17	0.17	0.8	n.a.		
p.Q218H	mut^0	0.04, 0.07	0.06	0.07, 0.11	0.09	0.4	n.a.		
p.N219Y	mut^0	0.21	0.21	0.24	0.24	1.2	n.a.		
p.Y231N	mut^-	0.03, 0.24, 0.46	0.25	9.35, 12.6, 14.5	12.2	60	4759, 7280	6020	1270
p.Y316C	mut^-	0, 0.03, 0.05, 0.17	0.06	3.38, 3.48, 3.70, 3.99	3.64	18	15.5, 24.1	19.8	4
p.L328F	mut^0	0.03, 0.06	0.05	0.04, 0.06	0.05	0.2	n.a.		
p.S344F	mut^-	0, 0.01, 0.05, 0.06	0.03	0.41, 0.54, 0.74, 0.79	0.62	3.1	27.9, 44.9	36.4	8
p.N366S	mut^-	0, 0.01, 0.07	0.03	0.49, 0.56, 0.71	0.59	2.9	219	219	46
p.R369H	mut^0	0.05, 0.16, 0.16	0.12	0.04, 0.28, 0.49	0.27	1.3	n.a.		
p.T387I	mut^-	0.01, 0.05, 0.37	0.14	1.06, 2.06, 2.39	1.84	9.1	66.6	66.6	14
p.G426R	mut^-	0, 0.22	0.11	2.39, 2.79	2.59	13	9261	9261	1954
p.F573S	mut^-	0, 0.01, 0.05, 0.07	0.03	0.93, 1.02, 1.05, 1.89	1.22	6.0	6.21, 36.5	21.4	5
p.P615T	mut^0	0.06, 0.24	0.15	0.10, 0.34	0.22	1.1	n.a.		
p.P615L	mut^0	0.07	0.07	0.09	0.09	0.4	n.a.		
p.V633G	mut^-	0, 0.02, 0.04	0.02	8.73, 9.99, 11.9	10.2	51	1016	1016	214
p.G648D	mut^-	0, 0.14	0.07	15.0, 19.4	17.2	85	6503, 6720	6612	1395
p.R694W	mut^-	0.01, 0.07, 0.19	0.09	0.40, 0.80, 0.99	0.73	3.6	45.9	45.9	10
p.R694L	mut^-	0.01, 0.02, 0.06	0.03	0.26, 0.67, 0.70	0.54	2.7	84.1	84.1	18
p.M700K	mut^-	0, 0.05, 0.05	0.03	0.60, 0.66, 0.75	0.67	3.3	543, 208	376	79
p.G717V	mut^-	0.01, 0.03, 0.06	0.03	2.78, 4.69, 8.88	5.45	27	8484	8484	1790
p.L736F	mut^-	0, 0.06, 0.20	0.08	1.54, 2.96, 3.47	2.66	13	221	221	47
wt	mean<br>range<br>n	0.46<br>0.01 - 2.94<br>10		20.2<br>12.4 - 34.1<br>10		100	4.74<br>1.65-12.3<br>7		1
vector only[#]	mean<br>range<br>n	0.04<br>0 – 0.17<br>7		0.06<br>0 – 0.25<br>7					

Transfection with mut^0 mutations resulted in severely deficient activities of both *holo*- and total MUT whereas mut^- mutations showed a clear response to AdoCbl with increase of MUT activity to variable levels associated with mild to highly elevated K_M for AdoCbl.

‘n.a.’, not applicable.
[¶]Values are single values or the mean of duplicates from replicate experiments.
[#]Negative control: MUT activities measured after transfection with empty pTracer vector.
